# Revisiting the standard for modeling the spread of infectious diseases

**DOI:** 10.1038/s41598-022-10185-0

**Published:** 2022-04-30

**Authors:** Michael Nikolaou

**Affiliations:** Chemical and Biomolecular Engineering Department, University of Houston, 4226 MLK Blvd, Houston, TX 77204-4004 USA

**Keywords:** Infectious diseases, Computational models

## Abstract

The COVID-19 epidemic brought to the forefront the value of mathematical modelling for infectious diseases as a guide to help manage a formidable challenge for human health. A standard dynamic model widely used for a spreading epidemic separates a population into compartments—each comprising individuals at a similar stage before, during, or after infection—and keeps track of the population fraction in each compartment over time, by balancing compartment loading, discharge, and accumulation rates. The standard model provides valuable insight into when an epidemic spreads or what fraction of a population will have been infected by the epidemic’s end. A subtle issue, however, with that model, is that it may misrepresent the peak of the infectious fraction of a population, the time to reach that peak, or the rate at which an epidemic spreads. This may compromise the model’s usability for tasks such as “Flattening the Curve” or other interventions for epidemic management. Here we develop an extension of the standard model’s structure, which retains the simplicity and insights of the standard model while avoiding the misrepresentation issues mentioned above. The proposed model relies on replacing a module of the standard model by a module resulting from Padé approximation in the Laplace domain. The Padé-approximation module would also be suitable for incorporation in the wide array of standard model variants used in epidemiology. This warrants a re-examination of the subject and could potentially impact model-based management of epidemics, development of software tools for practicing epidemiologists, and related educational resources.

## Introduction

The global epidemic of COVID-19 has brought to the forefront the importance of mathematical modelling in the development of strategies for managing the spread of infectious diseases^1–7^. Terms such as *flattening the curve*, ${R}_{0},$ or *herd immunity,* which entered public discourse^8^ emerge from mathematical models that purport to provide useful predictions and thus to help guide effective management strategies^9^. A basic class of such models separates a population into various compartments—each comprising individuals at a similar stage before, during, or after infection—and keeps track of the population fraction in each compartment over time, by balancing loading, discharge, and accumulation rates. The archetype for this modelling approach is the celebrated SIR model structure^10–17^ which splits a population into three compartments: susceptible (S) to the infection, infectious (I), and the rest (R) being immune or removed from infectious by recovery or death. The dynamics of how individuals move from S to I to R was developed almost a century ago in a mathematical modelling tour-de-force by Kermack and McKendrick^18^ who derived a general, if elaborate model structure in Eqs. (11)–(15) of their landmark paper. In the same publication (Eq. (29) *ibid.*) these authors also presented a well characterized special case in the form of the following three simple ordinary differential equations (ODEs) comprising the widely used standard SIR model:

1 $${s}^{{{\prime}}}(t)=-\beta s(t)i(t)$$

2 $${i}^{{{\prime}}}(t)=\beta s(t)i(t)-\gamma i(t)$$

3 $${r}^{{{\prime}}}(t)=\gamma i(t)$$

where $s,i,r$ are the susceptible, infectious, and removed fractions of a fixed-size population, respectively; $\beta , \gamma$ are infectivity and discharge constants, respectively; and each of the Eqs. (1)–(3) can be derived from the remaining two using the compatibility condition

4 $$s\left(t\right)+i\left(t\right)+r\left(t\right)=1$$

The great value of the SIR model is not merely that it can fit data (as already shown by Kermack and McKendrick in the same publication) but that it can also provide two deep and insightful conclusions about the dynamics governing the course of infectious disease epidemics. The first conclusion concerns the Threshold Theorem:

*…*there exists a critical or threshold density of population. *If the actual population density be equal to (or below) this threshold value the introduction of one (or more) infected person does not give rise to an epidemic, whereas if the population be only slightly more dense a small epidemic occurs* (ibid., p. 701)*.*

The second conclusion concerns the long-term behavior of $s, i, r$ at the asymptotic end of an epidemic:

*… the course of an epidemic is not necessarily terminated by the exhaustion of the susceptible members of the community. … the termination of an epidemic may result from a particular relation between the population density, and the infectivity, recovery, and death rates.* (ibid., pp. 701, 702, and Eq. (20))

**Figure 1:**
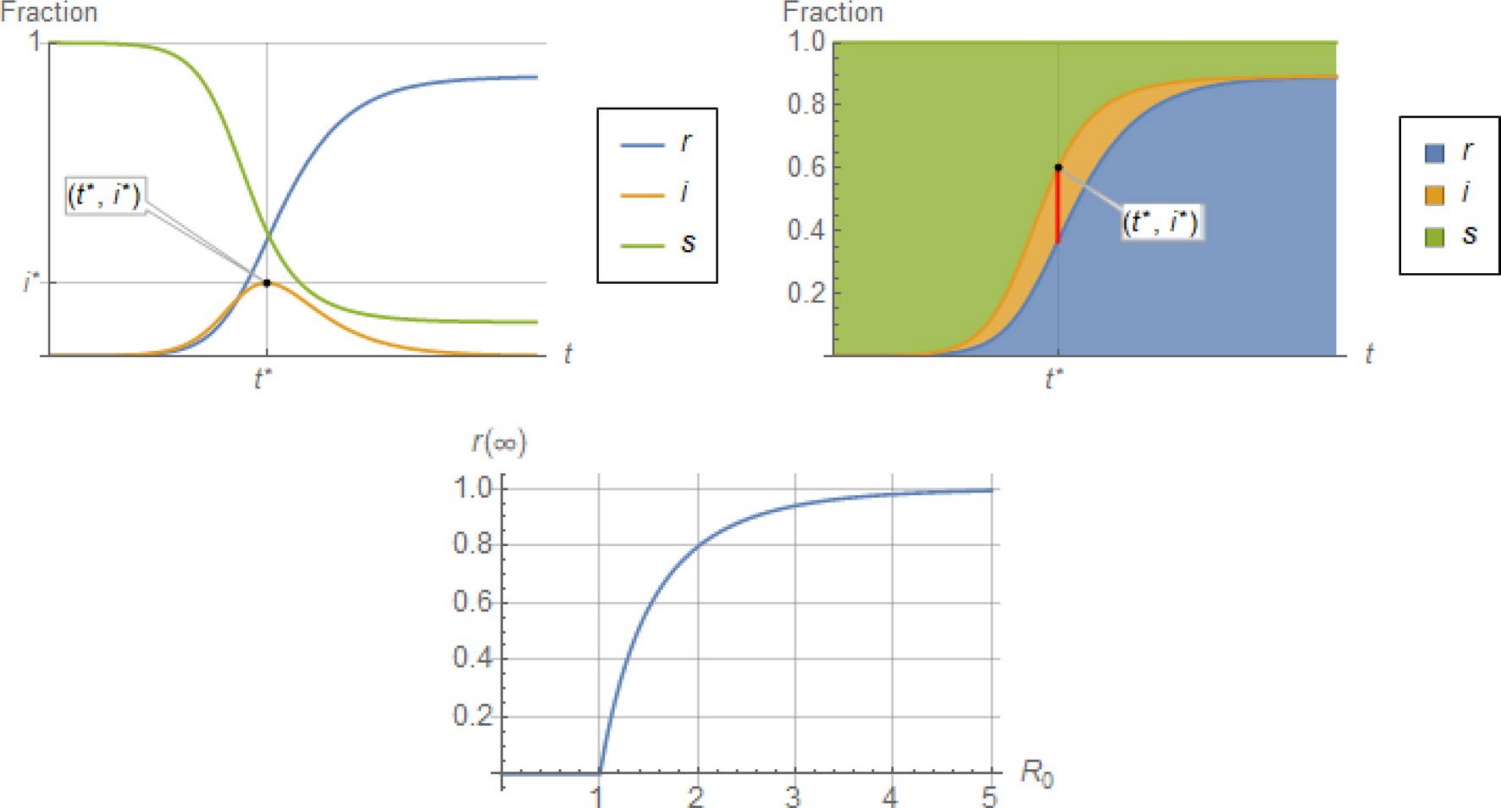
Top: Qualitative trends of individual (left) and stacked (right) profiles for $s, i, r$ fractions of a population in a spreading epidemic, from initiation to termination. Bottom: Total fraction of a population infected by the end of an epidemic, $r\left(\infty \right)=1-s\left(\infty \right),$ as a function of the basic reproductive ratio ${R}_{0}\stackrel{\scriptscriptstyle\mathrm{def}}=\frac{\beta }{\gamma },$ according to Eq. (6).

These conclusions are fairly robust, whether the general or the simplified version (Eqs. (1)–(3)) of the Kermack-McKendrick model is considered^15,18^. In fact, it immediately follows from stability analysis of Eqs. (1) and (2) that the threshold value for $s$ implied by the SIR model is

5 $${s}_{\mathrm{threshold}}=\frac{\gamma }{\beta }\stackrel{\scriptscriptstyle\mathrm{def}}=\frac{1}{{R}_{0}}$$

where ${R}_{0}$ (introduced as such later^15,19,20^) is the basic reproductive ratio, widely considered “one of the most critical epidemiological parameters”^11,21^. It also follows^18^ from Eqs. (1)–(3) that the total fraction of individuals infected throughout an epidemic, $r\left(\infty \right)$, is the real solution of the transcendental algebraic equation

6 $$\mathrm{ln}\frac{1-r\left(\infty \right)}{s\left(0\right)}+\left(1-r\left(\infty \right)-s\left(0\right)\right){R}_{0}=0$$

as depicted in Fig. 1. That figure shows the rapid escalation of $r\left(\infty \right)$ as ${R}_{0}$ rises above 1, given an initially susceptible population (In fact, making time dimensionless as $\eta \stackrel{\scriptscriptstyle\mathrm{def}}=\gamma t$, immediately transforms Eqs. (1) and (2) to ${s}^{{\prime}}\left(\eta \right)={R}_{0}s\left(\eta \right)i\left(\eta \right), {i}^{{\prime}}\left(\eta \right)=\left({R}_{0}s\left(\eta \right)-1\right)i\left(\eta \right)$, whose only parameter is ${R}_{0}$).

The above two quantitative predictions by Eqs. (5) and (6) lend exceptional value to the SIR model, both conceptually and computationally. For instance, they can be used to assess herd immunity^11^ for a population, corresponding to an estimated value of ${R}_{0}$ achieved by non-pharmaceutical or pharmaceutical interventions^22^. Or, conversely, for an epidemic that ran its course or in development, data can be used to gauge an overall or temporary value of ${R}_{0}$^21^.

However, as we will substantiate in the next section, there are another two important quantitative predictions of the standard SIR model that, we argue, can be problematic (see Fig. 1 for visualization):

- The *peak* value, ${i}^{*}$, of the infectious fraction, $i$, which may be misrepresented by as much as a factor of about 2, and
- the *exponential growth rate* of the infectious fraction, $i$, which is also misrepresented by as much as a factor of about 2, with corresponding misrepresentation of the *time to peak*, ${t}^{*}$.

The above two shortcomings are not confined to the standard SIR model but, as we elaborate in the next section, are far more pervasive and reach a wide area in compartment-based epidemiology modeling spanned by SIR variants.

To start with, good prediction of both the infectious peak, ${i}^{*}$, and the time to that peak, ${t}^{*}$, is of paramount importance when considering management strategies for an epidemic. This is because ${i}^{*}$ and ${t}^{*}$ significantly affect the resources needed for care of infectious patients. To wit, calls for *Flattening the Curve*^22^ during the COVID-19 epidemic aimed precisely at lowering ${i}^{*}$ and thus averting the overwhelming of medical care resources^23^.

In addition, good estimates of ${R}_{0}$ from data of exponential growth during the spread of an epidemic are critical for assessing the situation and for designing effective interventions^9,21^.

Furthermore, and more importantly, to the extent that predictions of the infectious peak and time-to-peak by the standard SIR model may be problematic, the problem is not confined to the I compartment of the standard SIR model. Rather, it may be endemic (no pun intended) in the numerous possible variants of compartment-based epidemiology models with loading and discharge terms. Such variants include a variety of compartments with corresponding arrangements and interactions (e.g., SEIR, SI, SIS, or similar^24^), multiple subpopulations (e.g., of different age and/or social contact structure^11,16,17,25^), spatial variation in addition to temporal (entailing partial differential equations^11^), and any combinations thereof, which collectively lead to diverse stratification patterns^26^. In the voluminous literature dealing with such models, the discharge rate from a compartment is *virtually always* represented by a term similar to the term $-\gamma i(t)$ of Eq. (2)^27^. In fact, this practice is so widespread in the entire literature of epidemiology^11,15,28–30^ that it is selected, perhaps uncritically, even in advanced modeling efforts which employ sophisticated tools (e.g., *automated algorithmic discovery*^31^) in attempts to uncover more realistic expressions for infection dynamics. It is plausible, therefore, to claim that peak and time-to-peak predictions for a related compartment in any of these models may be as problematic as the corresponding predictions of the simple SIR model, with similarly adverse consequences.

Of course, for more accurate predictions, one could forego the simplifying assumptions leading to Eqs. (1)–(3) and its variants, in favor of the general time-varying integrodifferential equation patterns introduced by Kermack and McKendrick^12,29,32^. This, however, would significantly increase complexity of analysis and use^32^, which partly explains the popularity and underscores the importance of simplified models such as SIR.

Consequently, a natural question arises: Given the aforementioned shortcomings of the standard SIR model, is there a mathematical model of comparable simplicity to Eqs. (1)–(3) that retains the two sound conclusions about the epidemic threshold (Eq. (5)) and long-term epidemic course (Eq. (6)) while avoiding the two issues mentioned above, namely misrepresentation of the infectious peak, ${i}^{*},$ and time to that peak, ${t}^{*}$?

Here, we constructively answer this question in the positive. Using a combination of Laplace transforms and Padé approximations to describe compartment discharge dynamics, we develop in the “The Padé SIR model structure” section (Eqs. (12) and (13)) of similar simplicity to SIR. The Padé SIR structure produces the exact same threshold and long-term values (Eqs. (5) and (6)) as Eqs. (1)–(3), while predicting more realistic infectious peak and time to peak for a wide range of practically significant cases. More importantly, because the proposed structure relies on replacement of the discharge term $-\gamma i(t)$ in the I module of the SIR Eq. (2) without increasing complexity, it can be used widely in the large array of compartment-based epidemiology models to realistically represent the dynamics of compartment discharge. This immediately prompts a re-evaluation and possible revision of the wide literature on compartment-based modeling in epidemiology inspired by the SIR model. It is emphasized that the preceding prompt is not motivated by a mere intent for higher accuracy; rather, the aim is to offer higher utility, in the spirit of George Box’s dictum “all models are wrong, but some are useful”^33^.

In the rest of the paper, we first present the significant merits and subtle issues of the SIR structure. Subsequently, we offer a remedy to these shortcomings, in the form of a new class of SIR variants (the Padé SIR model structure) whose properties and implications we explore for epidemiology modeling and epidemic management. Discussion and extensions follow, pointing to the usability of the proposed modeling approach and its applicability to the wide class of compartment-based epidemiology models.

## Methods

**Figure 2:**
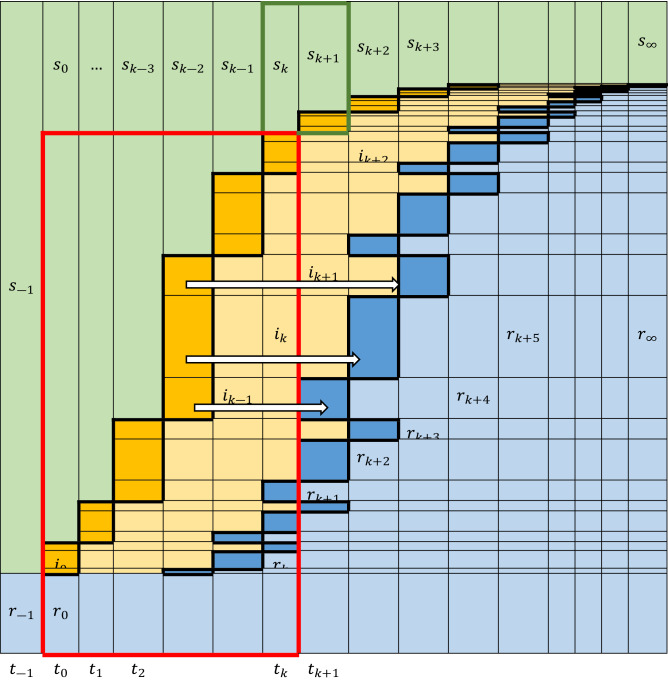
Schematic of time-varying susceptible ($s,$ green), infectious ($i,$ orange), and removed ($r,$ blue) fractions of a fixed-size population after an initial infection, ${i}_{0},$ at discretized time ${t}_{0}$. Each new part of the infectious fraction $i$ (thick-framed orange rectangles) moves to the removed fraction, $r$ (thick-framed blue rectangles) piecewise in a number of time steps following a certain distribution. The population eventually reaches a steady state at ${s}_{\infty }, {r}_{\infty }=1-{s}_{\infty },$ and ${i}_{\infty }=0$. The pattern analogy with the stacked chart in Fig. 1 is evident.

To provide context and intuition for the developments that follow, we will rely on the basic schematic of Fig. 2. Directly inspired by the original Kermack-McKendrick ideas, Fig. 2 shows how the stacked fractions $s,i,r$ of a fixed-size population change during an epidemic, as individuals move from compartment S to I to R over time. Infectious individuals in the I compartment are discharged (to enter the R compartment) at times $T\ge 0$ after becoming infectious, where the infectious period $T$ follows a cumulative distribution and corresponding density^18,29^ defined in the standard way as $\mathcal{F}\left(\theta \right)\stackrel{\scriptscriptstyle\mathrm{def}}=P\left[T\le \theta \right]$ and $\mathcal{f}\left(\theta \right)={\mathcal{F}}^{{\prime}}\left(\theta \right)$.

Simple balances around the boxed areas in Fig. 2 for a time-invariant cumulative distribution $\mathcal{F}\left(\theta \right)$ (APPENDIX A) yield the equation

7 $$r\left(t\right)=r\left(0\right)+{\int }_{0}^{t}\left(1-r\left(0\right)-s\left(t-\theta \right)\right)\underbrace{{{\mathcal{F}}^{{{\prime}}}\left(\theta \right)}}_{\mathcal{f}\left(\theta \right)}d\theta$$

which, combined with the infectivity Eq. (1) and the consistency Eq. (4) forms a general representation of the SIR system dynamics^29,32^.

Selecting $\mathcal{F}$ in Eq. (7) to be the cumulative density function of the exponential distribution $\mathcal{F}\left(\theta \right)=1-\mathrm{exp}\left(-\gamma \theta \right)\stackrel{\scriptscriptstyle\mathrm{def}}=F\left(\gamma \theta \right)$, one immediately gets the SIR model, Eqs. (1)–(3) (APPENDIX A). For that model, the parameter $\gamma$ is the inverse of

*the average infectious period … estimated relatively precisely from epidemiological data*^11^*.*

**Figure 3:**
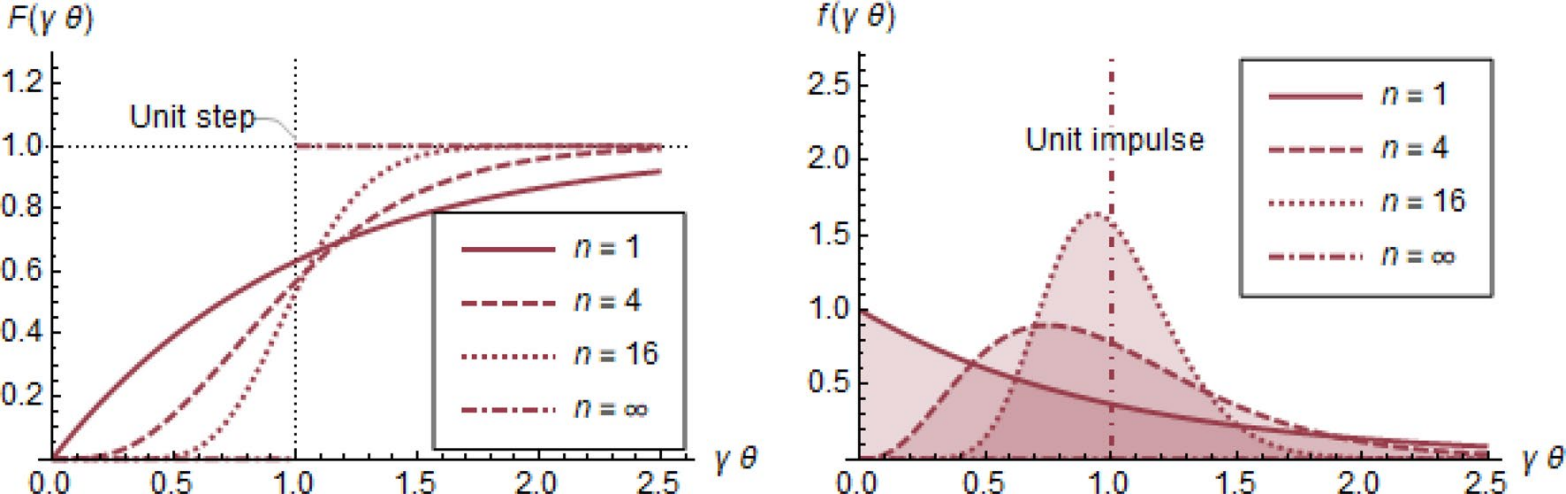
Sample cumulative distribution functions $F(\gamma \theta )$ (left) and corresponding probability distribution functions $f\left(\gamma \theta \right)={F}^{{\prime}}(\gamma \theta )$ (right) for discharge time, $T$, from the I compartment of a population. Curves follow the formulas $F\left(\gamma \theta \right)=1-\frac{\Gamma \left(n, n\gamma \theta \right)}{\Gamma (n, 0)}$ and $f\left(\gamma \theta \right)={F}^{{\prime}}\left(\gamma \theta \right),$ (see APPENDIX B). The exponential distribution with $F\left(\gamma \theta \right)=1-{e}^{-\gamma \theta }$ (left) and $f\left(\gamma \theta \right)={e}^{-\gamma \theta }$ (right) corresponds to $n=1$, whereas the impulse distribution with $F\left(\gamma \theta \right)=H(\gamma \theta -1)$ (unit step, left) and $f\left(\gamma \theta \right)=\delta \left(\gamma \theta -1\right)$ (unit impulse, right) correspond to $n=\infty$. All distributions are shown in terms of the dimensionless variable $\gamma \theta$ and have the same average equal to $1.$

As will be detailed in what follows, it is at this point where issues with peak and time-to-peak misrepresentations by the SIR model may originate:

Heeding the above suggestion to use epidemiological data for direct estimation of the average, $1/\gamma$, of the infectious period, $T$, is indeed sensible (in fact, necessary for a reasonable estimate). However, the associated distribution of $T$ is typically *far from exponential* (because an exponential distribution would suggest, inter alia, that most infectious individuals leave the I compartment in zero time, an untenable assumption). Rather, $T$ follows distributions with peak not near zero^34^ as shown in Fig. 3 by the curves indexed by $n\gg 1$. The exact shape of these curves is not important; rather, these curves serve as examples of distributions $f\left(\gamma \theta \right)$ with peak not near 0.

**Figure 4:**
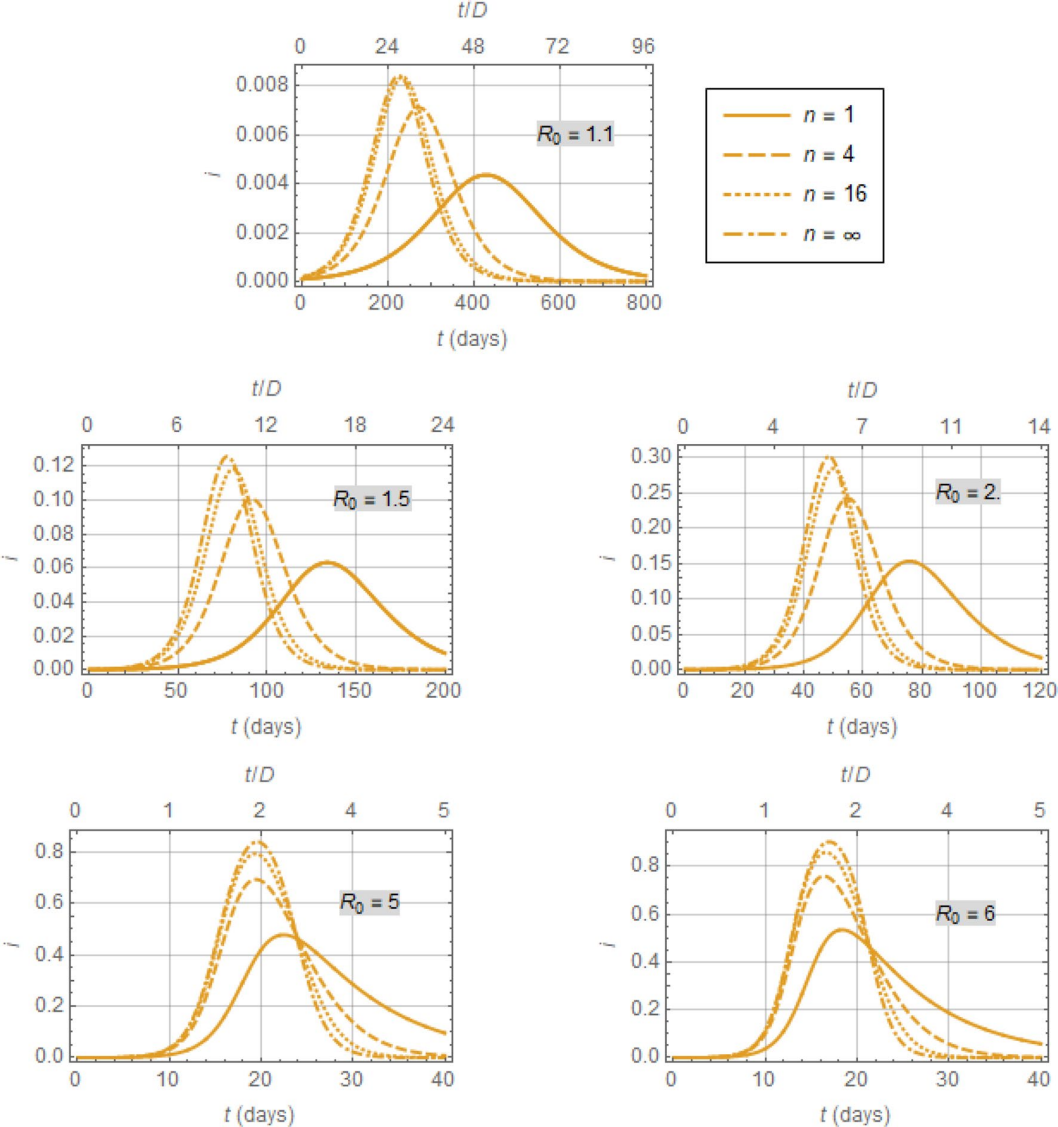
Response of the infectious fraction, $i(t)$, according to the model of Eqs. (1), (4), and (7), for distributions shown in Fig. 3. Note that the distribution for $n=4$ is closer to the exponential distribution $\left(n=1\right)$ than to the impulse distribution $(n=\infty )$ in Fig. 3, yet the response of $i(t)$ for $n=4$ is a lot closer to the response for $n=\infty$ rather than to that for $n=1.$

The assumption of exponential distribution for the infectious period “has appeared in many epidemic models but has seldom been questioned”^27^ yet would be conveniently acceptable, if it did not lead to inadvertent outcomes. Unfortunately it does, in the following subtle yet important way: While the *same* threshold and long-term values (Eqs. (5) and (6), respectively) would result from Eqs. (1), (7), and (4), and for practically *any* reasonable distribution of $T$ with the *same average,* $D\stackrel{\scriptscriptstyle\mathrm{def}}=1/\gamma$, (an insight already provided by Kermack and McKendrick^18^) the estimated *infectious peak* and *time to peak* would be significantly affected by the kind of distribution considered, in an interesting fashion, as demonstrated in Fig. 4. This figure shows the profiles of the infectious fraction, $i\left(t\right)$, for the infectious period distributions in Fig. 3, with time in both dimensional and dimensionless form. The latter is in terms of dimensionless time $t/D$, because this simple transformation trivially makes the dynamics of all considered models dependent on ${R}_{0}$ alone and allows for meaningful comparisons without loss of generality. The dimensional time is in days, to provide some context for epidemics such as COVID-19 with related values^34–36^

8 $$\frac{1}{\gamma }=8.4 \; \text{days}, \beta =\gamma {R}_{0} \; {\text{days}}^{-1}$$

What is remarkable in Fig. 4 is that while different distributions of $T$ sharing the same average, $D\stackrel{\scriptscriptstyle\mathrm{def}}=1/\gamma$, expectedly yield different profiles of $i(t)$^27^ these profiles *quickly approach the profile corresponding to the unit-impulse distribution* shown in Fig. 3. For that distribution of $T$, it immediately follows from Eqs. (1), (7), and (4) (APPENDIX A) that the resulting dynamic model, which we will term dSIR, comprises the delay differential equation (DDE)

9 $${s}^{{{\prime}}}\left(t\right)=\beta s\left(t\right)\left(s\left(t\right)-s\left(t-D\right)\right)$$

and the delay algebraic equations

10 $$i\left(t\right)=s\left(t-D\right)-s\left(t\right)$$

11 $$r\left(t\right)=1-s\left(t-D\right)$$

with $D\stackrel{\scriptscriptstyle\mathrm{def}}=\frac{1}{\gamma }$. Therefore, the dSIR model of Eqs. (9)–(11) constitutes a more realistic representation of spreading epidemic dynamics than the standard SIR model.

Delay differential equations (DDEs) such as the above have been a classic subject of study in biology^37,38^). DDEs are generally perceived as more difficult to analyze than ODEs^32^^, p. 5^ perhaps because of infinite spectra (for linear DDEs) or discontinuities in the derivatives of DDE solutions—albeit the corresponding theory for DDEs such as the above “does not present substantial additional difficulties” compared to ODEs^39, p]^^. 6^. Nevertheless, even though Eqs. (9)–(11) have long been known^29^ they are typically bypassed in favor of their ODE counterparts, Eqs. (1)–(3), along with their misrepresentations of the infectious peak and time to peak already discussed.

To address this issue, in the next section we derive *novel approximations* of the dSIR Eqs. (9)–(11) in the form of the *Padé SIR* ODEs, which have a number of advantages: While the Padé SIR model structure is as simple as that of the standard SIR model, Eqs. (1)–(3), and produces the same threshold and long-term values captured by Eqs. (5) and (6), it produces more realistic representations for the infectious peak and time to peak than the standard SIR ODEs. As such, the Padé SIR model structure not only creates an alternative to the standard SIR model but also provides a *general module that can be immediately incorporated in the wide variety of compartment-based models used in epidemiology*.

## Main results

### The Padé SIR model structure

Combining Laplace transforms with first-order Padé approximation (a popular approach for approximating transcendental transfer functions by polynomial rational fractions in automatic control^40,41^) one can show (APPENDIX C) that Eqs. (9)–(11) of the dSIR model can be approximated by the *first-order Padé SIR model*, comprising Eqs. (1), (4), and the novel ODE

12 $${i}^{{{\prime}}}\left(t\right)=\frac{2}{D}\left(\underbrace{{D\beta }}_{{R}_{0}}s\left(t\right)-1\right)i(t)$$

where $D$ is the value of the infectious period, $T.$ Note that the only difference between the above Eq. (12) and its standard SIR counterpart, Eq. (2), is simply the factor 2. Yet this difference has significant implications, to be highlighted shortly.

For better approximation of Eqs. (9)–(11) one can use a second-order Padé approximation to obtain (APPENDIX C) the *second-order Padé SIR model,* which comprises Eqs. (1), (4), and the second-order ODE

13 $${i}^{{{\prime}}{{\prime}}}\left(t\right)=\frac{12}{{D}^{2}}\left(\underbrace {{D\beta }}_{{R}_{0}} s(t)i(t)-i(t)-\frac{D}{2}{i}^{{{\prime}}}(t)\right)$$

in place of the SIR model’s Eq. (2).

### Why Padé SIR?

**Figure 5:**
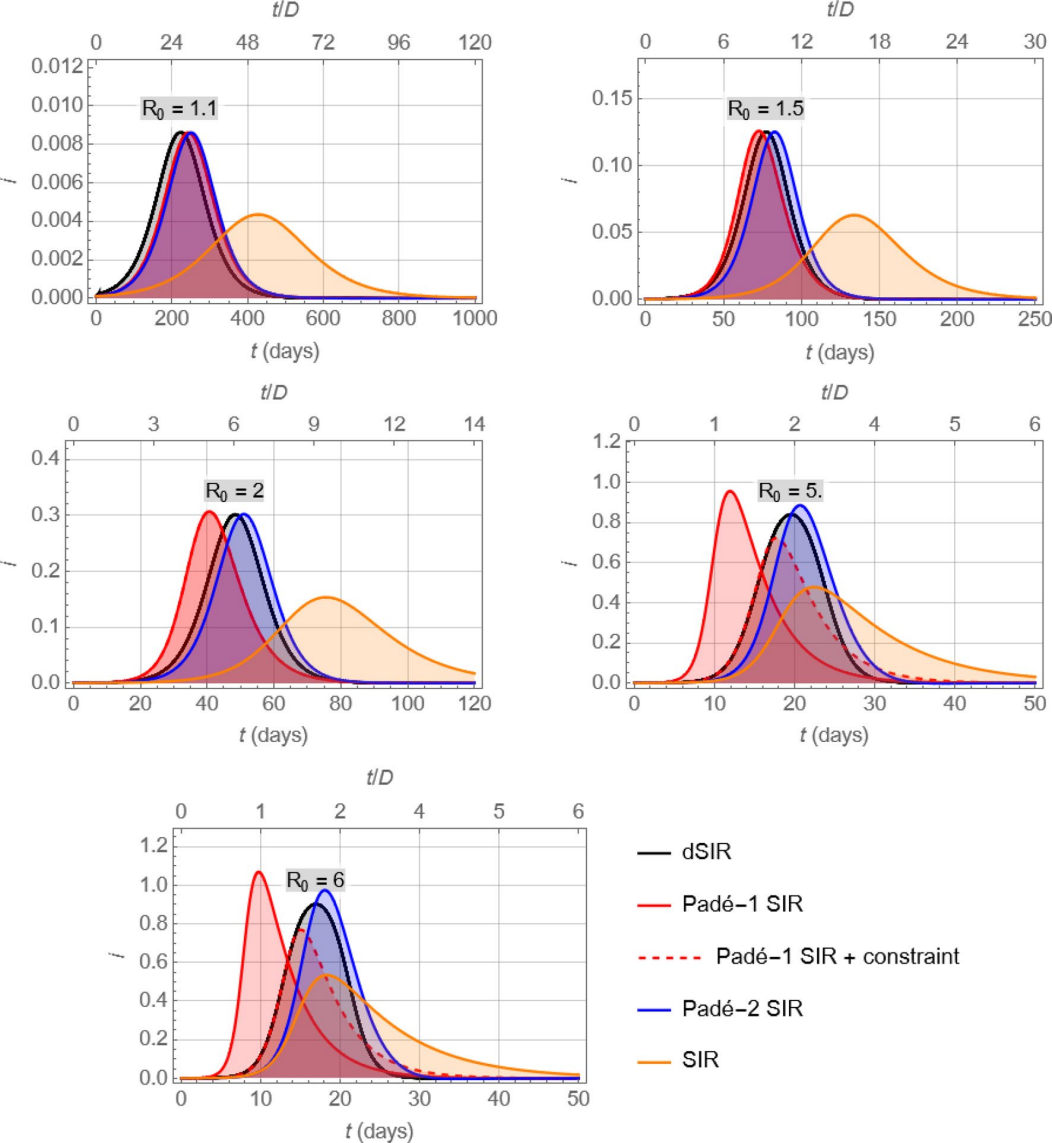
Comparison of the infectious fraction profiles, $i(t)$, resulting from the dSIR, Padé SIR (first- and second-order), and SIR models for different values of ${R}_{0}.$ Note that for relatively moderate values of ${R}_{0}$ both Padé SIR models approximate the dSIR model well, whereas for large values of ${R}_{0}$ the Padé-2 SIR remains a usable approximator while the Padé-1 SIR model approaches its usefulness limits.

A basic merit of the Padé SIR model is illustrated in Fig. 5, which shows that the profiles of $i(t)$ obtained by numerically integrating the (first- or second-order) Padé SIR models are close to that produced by the dSIR model for a range of values of ${R}_{0}$, but far from the corresponding profile produced by the standard SIR model.

Note that the approximation in Fig. 5 depends on ${R}_{0}\stackrel{\scriptscriptstyle\mathrm{def}}=\beta D$ and deteriorates as ${R}_{0}$ takes values farther away from 1, as expected by the properties of Padé approximants. In fact, the first-order Padé SIR model should be used with caution for ${R}_{0}\ge 2$, because it would yield negative early values of $r(t)$, as can be immediately deduced by linear analysis of the corresponding third ODE, ${r}^{{\prime}}\left(t\right)=-{s}^{{\prime}}\left(t\right)-{i}^{{\prime}}\left(t\right)=-\frac{{R}_{0}}{D}s\left(t\right)i\left(t\right)+\frac{2}{D}i\left(t\right)$, which implies $r\left(t\right)-\overline{r }\approx \frac{2-{R}_{0}}{D}i\left(t\right)$; and the same model, for larger ${R}_{0}\ge 2$, would produce peak values of $i\left(t\right)>1$, which is clearly meaningless. However, as shown in Fig. 5, the predictions of ${i}^{*}$ by the first-order Padé SIR remain remarkably close to those of the dSIR model, even for fairly large ${R}_{0}$ well above 2. This behavior of approximation accentuates the value of the Padé SIR model, as values of ${R}_{0}$ close to (or lower than) 1 would be far more desirable than values well above 1 (Fig. 1). Of course, one could easily extend the Padé SIR model to yield $r(t)$ values in the interval $\left[\mathrm{0,1}\right]$ through the simple modification ${r}^{{\prime}}\left(t\right)=\mathrm{max}\left(-\frac{{R}_{0}}{D}s\left(t\right)i\left(t\right)+\frac{2}{D}i\left(t\right), 0\right)$, as indicated for ${R}_{0}=5, 6$ in Fig. 5.

Figure 5 also shows profiles of $i\left(t\right)$ by the second-order Padé SIR model, and indicates that Padé approximations of third or higher order could be used in an similar way, but the point of diminishing returns would be quickly reached, as model complexity would increase a lot more quickly than quality of approximation.

Before discussing the important consequences implied by the Padé SIR model, relevant properties of that model are briefly summarized next, to better support the consequences established thereafter.

### Comparative summary of important properties of the Padé SIR models

The models considered can be analyzed using standard ODE or DDE theory, as already mentioned. Therefore, only aspects that bear insight or novelty will be discussed and corresponding comparisons will be made.

#### Instability at equilibrium and epidemic outbreak

It can be shown (APPENDIX D) that an equilibrium point, {$s = \bar s,\;i = 0,\;r = 1 - \bar s$} of the dSIR or of the Padé SIR model is stable and an epidemic outbreak does *not* occur iff $\overline{s }$ is below the threshold in Eq. (5). This result is in fact anticipated by the original Kermack and McKendrick analysis.

#### Final values of $\left\{s,i,r\right\}$

It can be shown (APPENDIX E) that at the end of an epidemic that started at $s\left(0\right)\approx \overline{s },$ $i\left(0\right)\approx 0,$ and $r\left(0\right)\approx 1-\overline{s },$ the total fraction of infected throughout the epidemic is

14 $$r\left(\infty \right)=1+\frac{W\left[-{R}_{0}s(0)\mathrm{exp}\left(-{R}_{0}s(0)\right)\right]}{{R}_{0}}$$

for all four models, where ${R}_{0}\stackrel{\scriptscriptstyle\mathrm{def}}=\beta D=\beta /\gamma$ and $W$ is the *Lambert function*^42^, whose importance in epidemiology modeling appears to have been recognized only recently^43^ (note that $\overline{s}\beta D\stackrel{\scriptscriptstyle\mathrm{def} }=\overline{s}{R }_{0}>1$ is required for the epidemic to spread). Equation (14) is the analytical solution of Eq. (6) and is precisely what is depicted in the graph of Fig. 1 for $s\left(0\right)\approx 1$.

#### Exponential rate of epidemic spread

**Figure 6:**
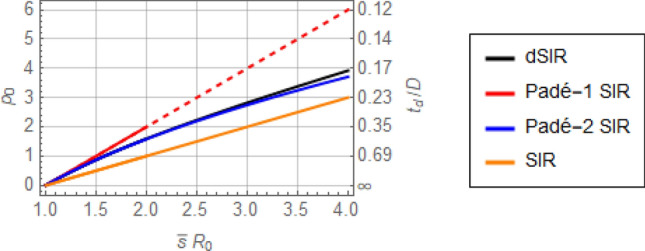
Dimensionless exponential rate,${\mathrm{p}}_{0}$, or doubling period, ${\mathrm{t}}_{\mathrm{d}}/\mathrm{D}$ (with respect to dimensionless time, $\eta =\gamma t\stackrel{\scriptscriptstyle\mathrm{def}}=t/D$) for the dSIR, Padé SIR, and SIR models, by Eqs. (17), (15), (16), and (18), respectively. The first three rates approach each other as $\overline{s}{R }_{0}$ approaches 1, whereas the SIR rate remains about half of the other two. The dashed portion of the Padé-1 SIR line is included only to indicate the trend, as the corresponding model would not be used in that range. Recall that ${p}_{0}=\mathit{ln}\left(2\right)D/{t}_{d}$ and that $\overline{s }=1$ for an epidemic with no prior immunity.

For the early part of a spreading epidemic, it can be shown (APPENDIX F) that the infectious fraction, $i(t)$, follows the approximately exponential growth

15 $$\frac{i\left(t\right)}{i\left(0\right)}\approx \mathrm{exp}\left(\underbrace {{2\left({R}_{0}\overline{s }-1\right)}} _{{p}_{0,\mathrm{ Pad}\acute{{\rm e}} -1\mathrm{ SIR}}} \underbrace {\frac{t}{D}}_ {\eta }\right)$$

16 $$\frac{i\left(t\right)}{i\left(0\right)}\approx \mathrm{exp}\left(\underbrace {{\left(-3+\sqrt{12{R}_{0}\overline{s }-3}\right)}} _ {{p}_{0,\mathrm{ Pad}\acute{{\rm e} }-2\mathrm{ SIR}}} \underbrace {\frac{t}{D}}_{\eta }\right)$$

according to the two Padé SIR models, or

17 $$\frac{i\left(t\right)}{i\left(0\right)}\approx a+b\,\,\mathrm{exp}\left(\underbrace {{\left(\overline{s}{R }_{0}+W\left[-\left(\overline{s}{R }_{0}\right){\mathrm{e}}^{-\overline{s}{R }_{0}}\right]\right)}}_{{p}_{0,\mathrm{ dSIR}}} \underbrace {\frac{t}{D}}_{\eta }\right)$$

according to the dSIR model, where the constants $a\ll b$ in Eq. (17) are in terms of ${R}_{0}\stackrel{\scriptscriptstyle\mathrm{def}}=\beta D$ (APPENDIX F). By comparison, the early growth of $i\left(t\right)$ according to the standard SIR model in Eqs. (1)–(3) is

18 $$\frac{i\left(t\right)}{i\left(0\right)}\approx \mathrm{exp}\left(\underbrace {{\left({R}_{0}\overline{s }-1\right)}}_{{p}_{0,\mathrm{SIR}}} \underbrace {{\gamma t}}_{\eta }\right)$$

where ${R}_{0}\stackrel{\scriptscriptstyle\mathrm{def}}=\beta /\gamma$. Note that in all four Eqs. (15)–(18) the rates ${p}_{0}$ depend on ${R}_{0}\overline{s }$ alone, as is anticipated by the corresponding dSIR, Padé SIR, and SIR models, for which introduction of the dimensionless time $\eta =\gamma t\stackrel{\scriptscriptstyle\mathrm{def}}=t/D$ leaves ${R}_{0}$ as the only remaining parameter in the corresponding equations. Therefore, the above exponential rates ${p}_{0}$ are shown as functions of $\overline{s}{R }_{0}$ in Fig. 6. Note that $\overline{s }=1$ for an epidemic without prior immunity in the population.

It is evident in Fig. 6 that the rates (or doubling periods) corresponding to the Padé SIR and dSIR models differ from their SIR counterparts by a factor of about 2, for $\overline{s}{R }_{0}$ not much higher than 1. This agrees well with the more rapid early rise of $i(t)$ from numerical integration of the dSIR and Padé SIR models compared to that of the SIR model, as shown in Fig. 5. Note again that despite these rate differences shown in Fig. 6, all four models considered eventually reach the same steady-state values, as captured by Eq. (14).

The importance of these discrepancies for estimation of ${R}_{0}$ from early epidemic data will made clear shortly.

#### Peak of infectious fraction

While an analytical solution for ${i}^{*}$ according to the dSIR model is not obvious to the author, a good approximation can be easily obtained (APPENDIX G) through the first-order Padé SIR model, following the same approach taken for the SIR model, to get

19 $${i}_{\mathrm{Pad}{\acute{{\rm e}} }-1\mathrm{ SIR}}^{*}=2\left(s\left(0\right)-\frac{\mathrm{ln}\left({R}_{0}s\left(0\right)\right)}{{R}_{0}}-\frac{1}{{R}_{0}}\right)$$

The above ${i}^{*}$, for the same ${R}_{0}$, is exactly double the ${i}^{*}$ of the standard SIR model, which is known to be

20 $${i}_{\mathrm{SIR}}^{*}=s\left(0\right)-\frac{\mathrm{ln}\left({R}_{0}s\left(0\right)\right)}{{R}_{0}}-\frac{1}{{R}_{0}}$$

(APPENDIX G). This discrepancy accounts for the differences observed in Fig. 5 between the ${i}^{*}$ produced by the SIR and by the other three models considered. Obviously, this approximation breaks down for values of ${R}_{0}$ that yield ${i}_{\mathrm{Pad}\acute{{\rm e}}-1\mathrm{ SIR}}^{*}>1$, a situation that would be expected for large values of ${R}_{0}$, as illustrated in the last plot $\left({R}_{0}=6\right)$ of Fig. 5.

The discrepancy between the SIR and Padé SIR models also manifests itself in using them for model-based predictions that depend on parameter estimates driven by epidemiological data, as discussed in the next section.

## Discussion and extensions

### Model-based predictions form fitting epidemiological data

An immediate and important discrepancy for the models discussed is in the estimation of ${R}_{0}$ from epidemiological data on daily new cases during exponential growth, i.e. from $i(t)$ or $i{^{\prime}}(t)$, and from the average infectious period, $D\stackrel{\scriptscriptstyle\mathrm{def}}=1/\gamma$. Figure 6 captures the relationship between the exponential growth rate ${p}_{0}$ given a corresponding $\overline{s}{R }_{0}$. Therefore, for $s\left(t\right)\approx \overline{s }=1$, it is standard to use a simple log-plot of daily new cases vs. time to estimate the slope ${p}_{0}=\mathrm{ln}\left(2\right)D/{t}_{d}$ of $\mathrm{exp}\left({p}_{0}t/D\right)$ (where ${t}_{d}$ is the doubling period) and from that the resulting ${R}_{0}$. Following this procedure for $\overline{s }=1$ (no prior immunity) the two Padé SIR models, Eqs. (15) and (16), yield the novel ${R}_{0}$ estimates

21 $${R}_{0,{\mathrm{Pad}}\acute{{\rm e}}-1\mathrm{ SIR}}=\frac{{p}_{0}}{2}+1$$

22 $${R}_{0,{\mathrm{Pad}}\acute{{\rm e}}-2\mathrm{ SIR}}=\frac{{\left({p}_{0}+3\right)}^{2}+3}{12}$$

the dSIR model yields

23 $${R}_{0,\mathrm{ dSIR}}=\frac{{p}_{0}}{1-\mathrm{exp}\left(-{p}_{0}\right)}$$

whereas the standard SIR model, Eq. (2), yields the well known estimate^9,21,44^

24 $${R}_{0,\mathrm{ SIR}}={p}_{0}+1$$

The above Eqs. (21)–(24) can be visualized in Fig. 6 with ${p}_{0}$ considered the independent variable. Note that ${R}_{0, \mathrm{ Pad}\acute{{\rm e}}-1\mathrm{ SIR}}-1=\frac{{R}_{0, \mathrm{ SIR}}-1}{2}$ and ${R}_{0, \mathrm{ Pad}\acute{{\rm e}}-2\mathrm{ SIR}}=\frac{{R}_{0, \mathrm{ SIR}}-1}{2}\left(1+\frac{{R}_{0, \mathrm{ SIR}}-1}{6}\right)$ and that ${R}_{0, \mathrm{dSIR}}\approx {R}_{0, \mathrm{ Pad}\acute{{\rm e}}-1\mathrm{ SIR}}$ for small ${p}_{0}$.

The important message of Fig. 6 is that systematic error may arise in the estimation of ${R}_{0}$ when using the standard SIR model. For example, taking $D=8.4\mathrm{ days}$ (Eq. (8) for COVID-19) and ${t}_{d}=2.3 \; \mathrm{days}$ (corresponding to early COVID-19 spread in the US^45^) yields ${R}_{0,\mathrm{dSIR}}\approx {R}_{0,\mathrm{Pade}-2\mathrm{ SIR}}=3.2$ vs. ${R}_{0,\mathrm{SIR}}=4$ for $\frac{{t}_{d}}{D}=0.23$ in Fig. 6. As ${t}_{d}$ increases, the discrepancy between ${R}_{0,\mathrm{dSIR}}$ or ${R}_{0,\mathrm{Pade}-2\mathrm{ SIR}}$ on one hand and ${R}_{0,\mathrm{SIR}}$ on the other becomes more pronounced.

**Figure 7:**
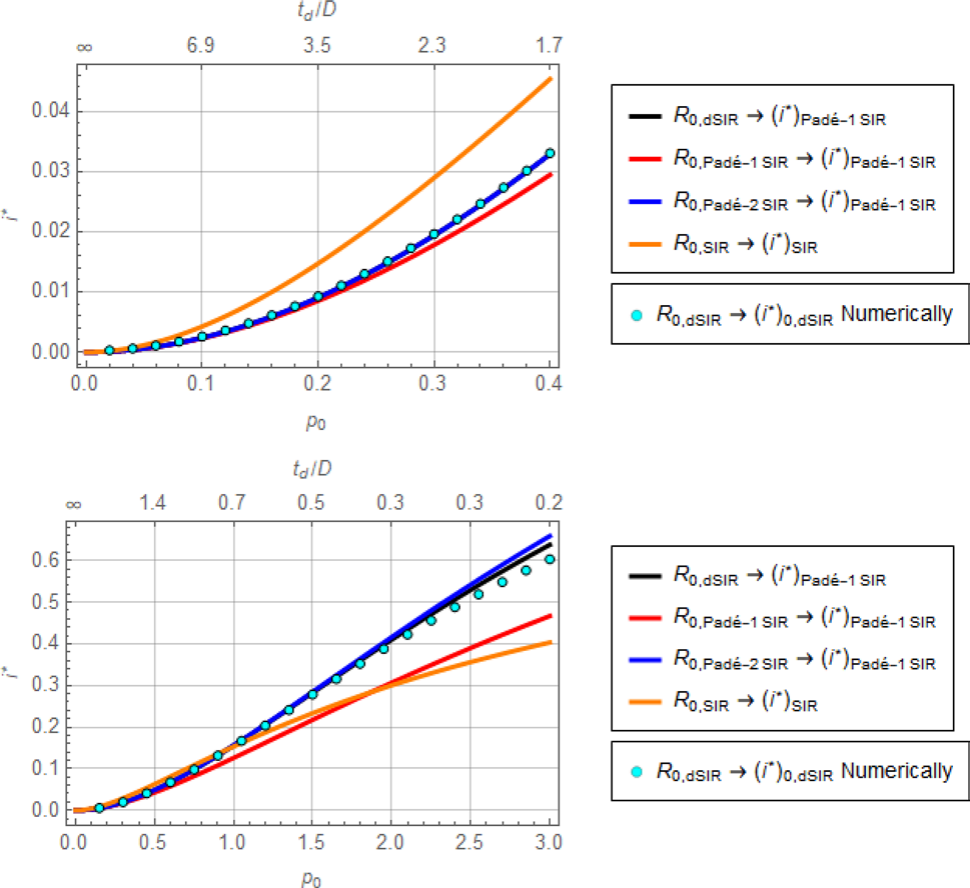
Predicted maximum infectious fraction, ${i}^{*}$, based on the exponential rate, ${p}_{0}$, of an epidemic spread. The values of ${i}^{*}$ are calculated by (**a**) the analytical expression of the Pade-1 SIR model fed with estimates of ${R}_{0}$ from ${p}_{0}$ according to the dSIR, Pade-1 SIR, and Pade-2 SIR models, (**b**) the analytical expression of the standard SIR model fed with an estimate of ${R}_{0}$ from ${p}_{0}$ according to the same model, and (**c**) numerically by integration of the dSIR model fed with an estimate of ${R}_{0}$ from ${p}_{0}$ according to the same model. The top graph is portion of the bottom graph at higher resolution.

Systematic errors in estimates of ${R}_{0}$ have important implications. For example, the conceptual anticipation of total infected through the pandemic, as shown in Fig. 1, following Eq. (14), is going to be significantly affected. In addition, the infectious peak is also going to be affected in a non-trivial way, as shown in Fig. 7. In that figure, profiles of ${i}^{*}$ are plotted as functions of the exponential growth rate, ${p}_{0}$, through the following procedure: Given ${p}_{0}$, the corresponding values of ${R}_{0}$ are computed according to the dSIR, Padé-1 SIR, Padé-2 SIR, and SIR models (Eqs. (21)–(24)) and, subsequently, values of ${i}^{*}$ are computed using Eq. (19) (Padé-1 SIR model) for the first three ${R}_{0}$ values and Eq. (20) for the fourth value of ${R}_{0}$. For calibration, the dots in Fig. 7 represent calculation of ${i}^{*}$ through direct numerical integration of the dSIR Eqs. (9)–(11) for values of ${R}_{0}$ computed using Eq. (23). There is remarkable closeness of ${i}^{*}$ values produced by the Padé SIR models to the ideal values produced by the dSIR model, contrasted to the distance of ${i}^{*}$ values produced by the standard SIR model.

The message from this exercise is that although adjusting the parameter ${R}_{0}$ of the standard SIR model can fit data from exponential epidemic growth well, there will remain two significant problems, namely neither the estimated ${R}_{0}$ nor the predicted ${i}^{*}$ will be represented well. The proposed model structures offer a better representation.

### Analytical calculation of ${R}_{0}$ to observe an upper bound on ${i}^{*}$

Of practical interest is the situation where an upper bound is placed on ${i}^{*},$ to avoid the overwhelming of hospitalization facilities during an epidemic. For that situation, Eq. (19) of the Padé-1 SIR model has an explicit analytical solution for the corresponding ${R}_{0}\stackrel{\scriptscriptstyle\mathrm{def}}=\beta D$ as

25 $$s(0)\beta D\stackrel{\scriptscriptstyle\mathrm{def}}=s(0){R}_{0}=\frac{2{W}_{-1}\left(\frac{{i}^{*}/s(0)-2}{2\mathrm{e}}\right)}{{i}^{*}/s(0)-2}$$

where ${W}_{-1}$ is the Lambert function of order $-1$ and typically $s\left(0\right)\approx 1$ without prior immunity. By comparison, the standard SIR model yields

26 $$s(0)\frac{\beta }{\gamma }\stackrel{\scriptscriptstyle\mathrm{def}}=s(0){R}_{0}=\frac{{W}_{-1}\left(\frac{{i}^{*}/s(0)-1}{\mathrm{e}}\right)}{{i}^{*}/s(0)-1}$$

**Figure 8:**
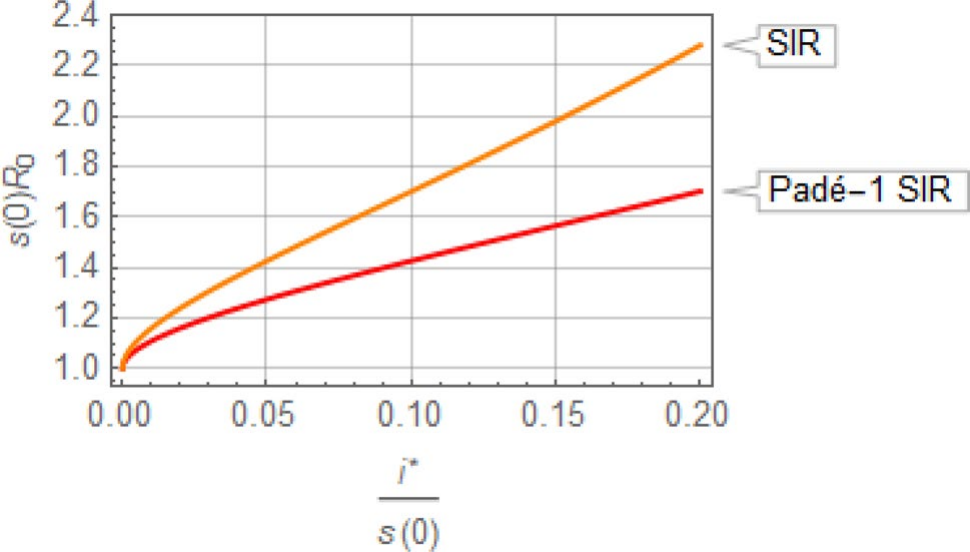
Maximum value of ${R}_{0}$ indicated by the Padé SIR and SIR models for $\mathrm{i}$ not to exceed ${i}^{*}$ when $s\left(0\right)\approx 1.$

The values of ${R}_{0}$ indicated by Eqs. (25) and (26), with corresponding definitions, are shown in Fig. 8. It is evident that the Padé SIR model places twice as tight a restriction on $\left({R}_{0}-1\right)$ as the standard SIR model, if $i$ is not to exceed the specified ${i}^{*}$ value. The implications of this result for tasks such as *Flattening the Curve* through interventions that adjust ${R}_{0}$ are clear.

### How does the Padé SIR model work?

**Figure 9:**
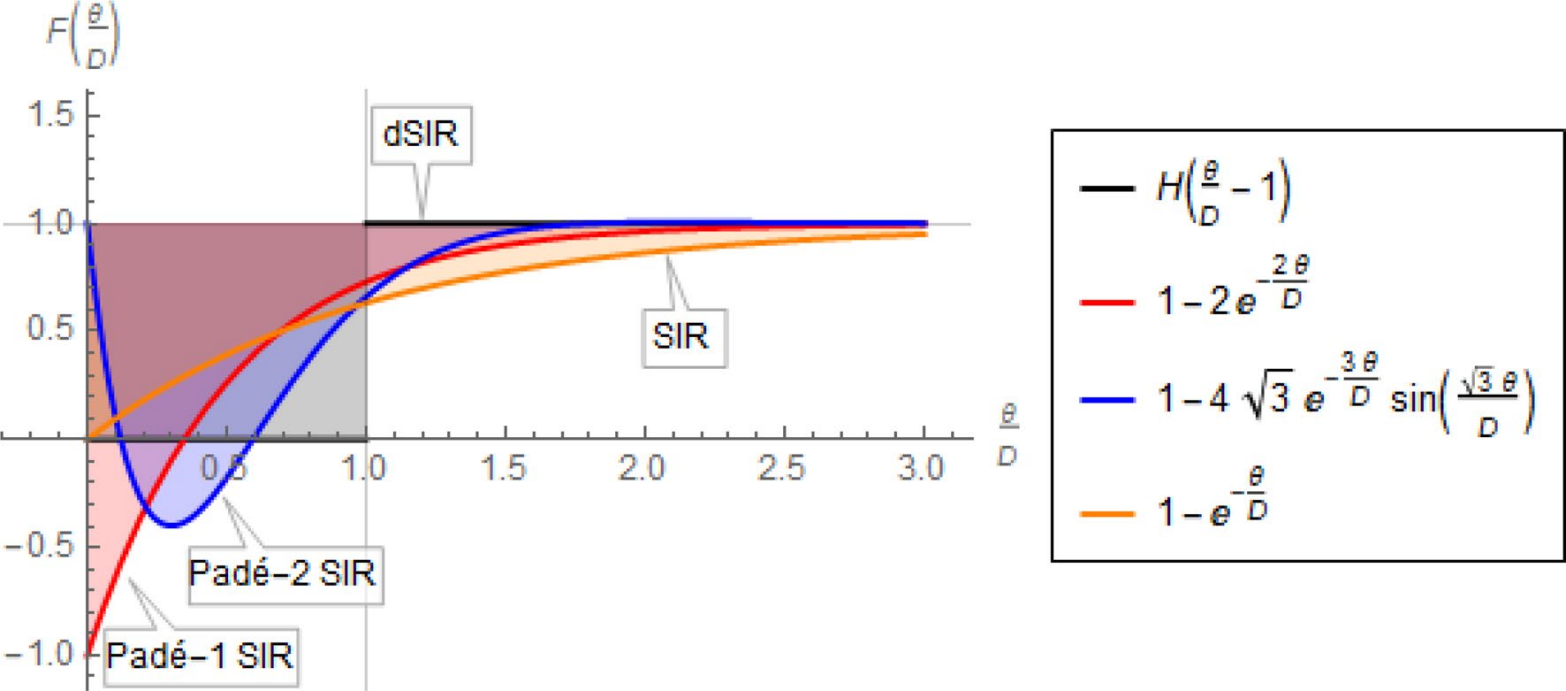
Distributions $F\left(\frac{\theta }{D}\right)$ of the dimensionless infectious period $\theta /D$ with average $1.$ The curves shown imply that the newly infected are removed from the infectious compartment, I, according to the formulas shown (cf. Fig. 5). All distributions have the same average equal to 1, as also indicated by the shaded areas.

Underlying the Padé SIR model are constructs for approximating the unit-step cumulative distribution of the infectious time period, $T$, shown in Fig. 3$(n=\infty )$, as explained in APPENDIX C. Graphs of these approximations and their corresponding formulas are presented in Fig. 9, along with the exponential and unit-step distributions for comparison. Note that the two Padé SIR distributions in Fig. 9 might appear absurd, as they involve negative values. However, this pattern turns out to yield acceptable values for the fractions $s,i,r$.

**Figure 10:**
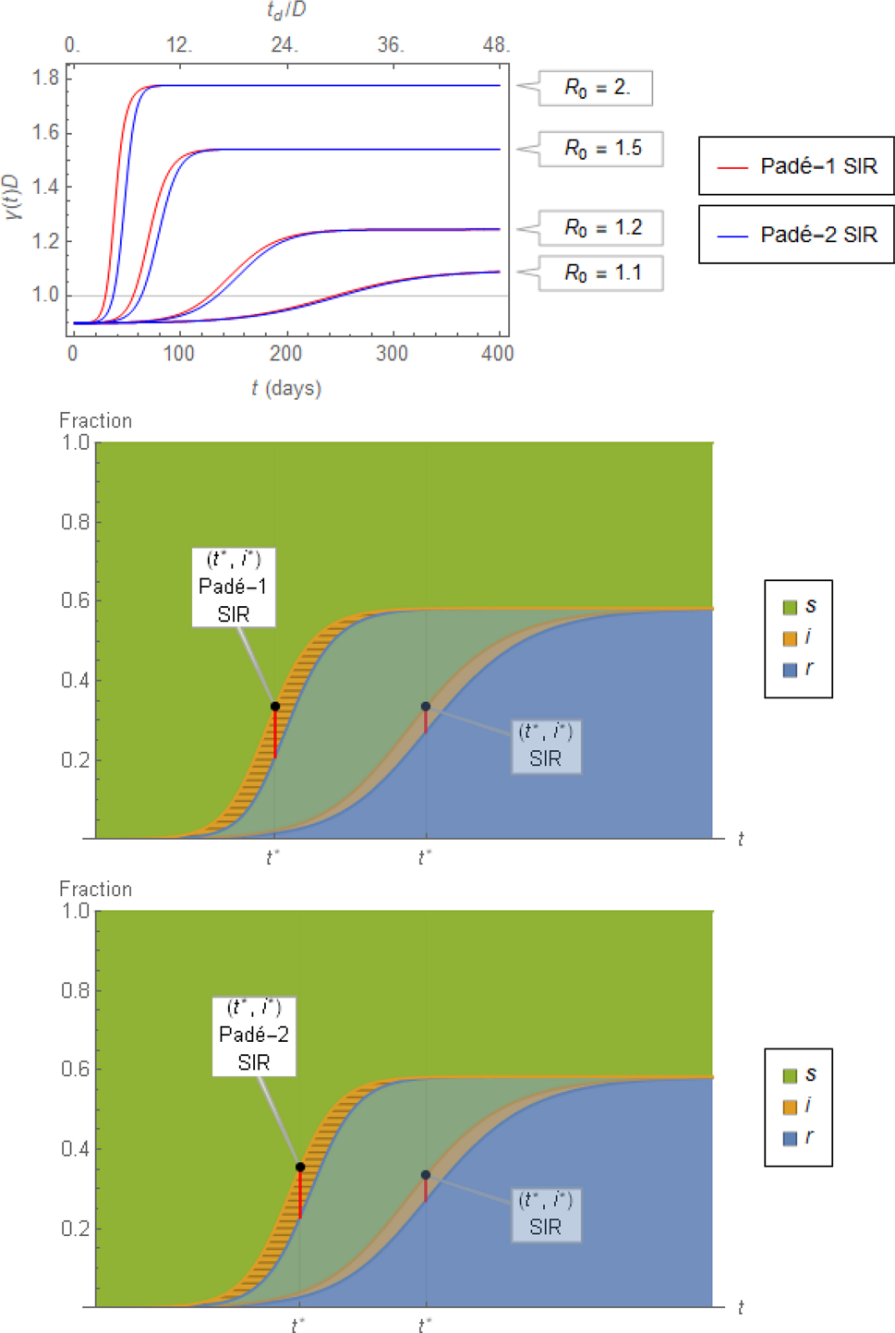
Top: Comparison between the inferred time-varying $\gamma \left(t\right)\stackrel{\scriptscriptstyle\mathrm{def}}={r}^{{\prime}}(t)/i(t)$ and the corresponding time-invariant $1/D=0.12 {day}^{-1}$ for various ${R}_{0}\stackrel{\scriptscriptstyle\mathrm{def}}=\beta D$ *i*n numerical integration of the first- $({r}^{{\prime}}(t)/i(t) =\beta s(t)-2/D)$ and second-order $({r}^{{\prime}}(t)/i(t) =\beta s(t)-{i}^{{\prime}}(t)/i(t)$*)* Padé SIR equations. Middle and bottom: Stacked fractions $\left\{s,i,r\right\}$ of* a* population through an epidemic for ${R}_{0}=1.5$ according to the first- and second-order Padé SIR model, superimposed on the standard SIR model (cf. Figure 1). The horizontal slices of equal length $D$ shown in the orange area for $i(t)$ are the continuous counterparts of the same area in the discretized plot of Fig. 2.

It should also be noted that Eq. (12) of the first-order Padé SIR suggests that the infectious loading rate remains $\frac{{R}_{0}}{D}s\left(t\right)i\left(t\right)$, whereas the infectious discharge rate appears as $\frac{{R}_{0}s\left(t\right)-2}{D}i(t)$ rather than $-i(t)/D$, suggested by Eq. (2). This is illustrated visually in Fig. 10 in two ways, both of which underscore the significant differences between the SIR model on one hand and dSIR and Padé SIR models on the other: First (top), a time-varying $\gamma \left(t\right)\stackrel{\scriptscriptstyle\mathrm{def}}=r {^{\prime}}(t)/i(t)$ (following Eq. (3)) is shown, with the values of ${r}^{{\prime}}\left(t\right)$ and $i(t)$ calculated by the first- or second-order Padé SIR model with a fixed $D$. Note that the discrepancy between $D$ and $1/\gamma \left(t\right)$ (shown as values of $\gamma \left(t\right)D$ in Fig. 10) remains appreciable even for values of ${R}_{0}$ close to 1. Second (middle and bottom), Fig. 10 shows in a stacked plot the differences between the fractions $\left\{s\left(t\right),i\left(t\right),r\left(t\right)\right\}$ produced by the (first- or second-order) Padé SIR models and the SIR model. In addition to the clear difference in the time profiles and infectious fraction peaks, note that the horizontal slices of the orange segments, corresponding to the infectious period for each newly infected fraction (Fig. 2), remain constant (equal to $D$) over time for the Padé SIR model, in contrast to the SIR model, for which the infectious period increases (Fig. 10, top).

### The proposed approach in the context of Kermack and McKendrick

In the sentence right before they present their SIR model in Equ. (29) of their paper, Kermack and McKendrick^18^ explain that this is a

*special case in which* $\phi$ *and* $\psi$ *are constants* $\kappa$ *and* $l$ *respectively*.

with $\left(\kappa , l\right)$ refering to $\left(\beta ,\gamma \right)$ of Eqs. (1)–(3), respectively. The assumption about constant $\phi$ is plausible, as it refers to the rate of spread of the epidemic (cf. Eq. (1)). While that parameter might change over time as a result of interventions taken to curb an epidemic, such changes could easily be reflected in the SIR model by a time-varying $\phi$ (cf. $\beta$ in Eqs. (1) and (2)). The assumption about constant $\psi ,$ however, as widely as it may have been used, is chosen for mathematical convenience rather than for reasonableness of representation:

*If* ${\psi }_{\theta }$ *denotes the rate of removal, …, then the number who are removed from each* $\theta$ *group at the end of the interval* $t$ *is* ${\psi }_{\theta }{v}_{t,\theta }$*, (ibid., p. 703).*

where

${v}_{t,\theta }$ *shall denote the number of individuals in unit area at the time* $t$ *who have been infected for* $\theta$ *intervals (ibid., p. 702).*

However, the rate of removal depends more on the duration over which individuals have remained infected and less on the size of that group. It is this simple fact that is critiqued here and alternatives for which are proposed.

Finally, it is fitting to quote Kermack and MacKendrick’s remarks on fitting field data from a plague outbreak: Along with using the SIR model, thereby assuming an exponential distribution of infectious time after infection, these authors explicitly state five additional simplifying assumptions (p. 715, *ibid.*) and warn that

*deductions as to the actual values of the various constants should not be drawn. It may be said, however, that the calculated curve, …, conforms roughly to the observed figures.*

Indeed, all four models considered in this study (dSIR, 1-/2- Padé SIR, and SIR) fit well the data mentioned. Yet, were these models to be used for fitting the early exponential spread of the epidemic, their projections would be quite different, as already elaborated on.

### Extensions

As already mentioned, the proposed approach to compartment-based epidemiological modeling is applicable to model structures with a variety of compartments and flows among them. For these structures, the corresponding ODE models resulting from compartment discharge rates proportional to the load of each corresponding compartment^14^ can be immediately translated (a) from ODEs to DDEs with each compartment delay equal to the average residence time of that compartment, and (b) from DDEs to (first- or second-order) Padé approximations, which retain an ODE structure.

To substantiate these claims by an example, we briefly discuss next an extension of the ideas developed for the SIR structure to the SPIR variant that includes a compartment P between S and I (APPENDIX H). Individuals in the P compartment (equivalent to the E compartment in the standard SEIR structure^10,11,46,47^) are asymptomatic infectious, that is they can transmit the disease before they enter the I compartment as symptomatic infectious^48^ a trait observed in several occasions, notably in the current COVID-19 epidemic^23,49,50^. Corresponding equations are shown in Table 1.

**Table 1:** Equations for SPIR, dSPIR, Padé-1 SPIR and Padé-2 SPIR models.

${s}^{{{\prime}}}\left(t\right)=-{\beta }_{i}s\left(t\right)i\left(t\right)-{\beta }_{p}s\left(t\right)p\left(t\right)$ ${p}^{{{\prime}}}\left(t\right)={\beta }_{i}s\left(t\right)i\left(t\right)+{\beta }_{p}s\left(t\right)p\left(t\right)-\alpha p\left(t\right)$ ${i}^{{{\prime}}}\left(t\right)=\alpha p\left(t\right)-\gamma i\left(t\right)$ ${r}^{{{\prime}}}\left(t\right)=\gamma i\left(t\right)$	${s}^{{{\prime}}}\left(t\right)=-{\beta }_{i}s\left(t\right)i\left(t\right)-{\beta }_{p}s\left(t\right)p\left(t\right)$ $p\left(t\right)=s\left(t{-D}_{p}\right)-s\left(t\right)$ $i\left(t\right)=s\left(t{-D}_{i}\right)-s\left(t{-D}_{p}\right)=$ $=s\left(t{-D}_{i}\right)-s\left(t\right)-p\left(t\right)$ $r\left(t\right)=1-s\left(t{-D}_{i}\right)$
${s}^{{{\prime}}}\left(t\right)=-{\beta }_{i}s\left(t\right)i\left(t\right)-{\beta }_{p}s\left(t\right)p\left(t\right)$ ${p}^{{{\prime}}}\left(t\right)=2\left({\beta }_{i}s\left(t\right)i\left(t\right)+{\beta }_{p}s\left(t\right)p\left(t\right)-\frac{p\left(t\right)}{{D}_{p}}\right)$ ${i}^{{{\prime}}}\left(t\right)=2\left(\left(\frac{1}{{D}_{p}}-\frac{1}{{D}_{i}}\right)p\left(t\right)-\frac{1}{{D}_{i}}i\left(t\right)\right)$ $r\left(t\right)=1-s\left(t\right)-p\left(t\right)-i\left(t\right)$	
${s}^{{{\prime}}}\left(t\right)=-{\beta }_{i}s\left(t\right)i\left(t\right)-{\beta }_{p}s\left(t\right)p\left(t\right)$ ${p}^{{{\prime}}{{\prime}}}\left(t\right)=\frac{12}{{D}_{p}^{2}}\left(\underbrace{{{D}_{p}{\beta }_{p}}}_{{R}_{0p}}s\left(t\right)i\left(t\right)-p\left(t\right)-\frac{{D}_{p}}{2}{p}^{{{\prime}}}\left(t\right)\right)$ ${i}^{{{\prime}}{{\prime}}}\left(t\right)=-{p}^{{{\prime}}{{\prime}}}\left(t\right)+\frac{12}{{D}_{i}^{2}}\left(\underbrace {{{D}_{i}{\beta }_{i}}} _{{R}_{0i}} s\left(t\right)i\left(t\right)-i\left(t\right)-p\left(t\right)-\frac{{D}_{i}}{2}\left({i}^{{{\prime}}}\left(t\right)+{p}^{{{\prime}}}\left(t\right)\right)\right)$ $r\left(t\right)=1-s\left(t\right)-p\left(t\right)-i\left(t\right)$	

Note the correspondence ${D}_{p}\stackrel{\scriptscriptstyle\mathrm{def}}=\frac{1}{\alpha }, {D}_{i}\stackrel{\scriptscriptstyle\mathrm{def}}=\frac{1}{\alpha }+\frac{1}{\gamma }>{D}_{p}$. Also note that if ${\beta }_{i}={\beta }_{p}$, treating $p\left(t\right)+i(t)$ as a single variable renders the SPIR structures similar to the SIR structures with similar dynamics.

**Figure 11:**
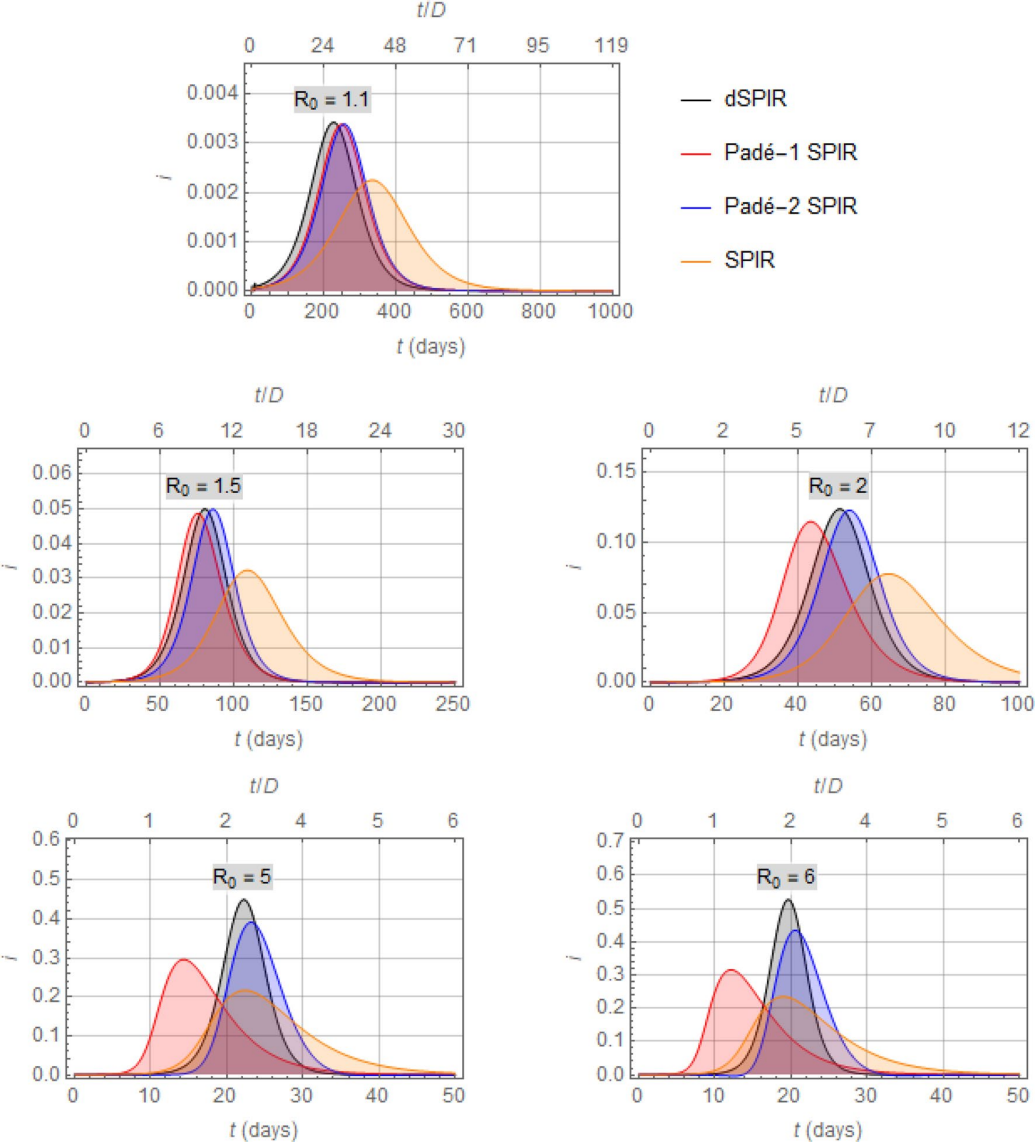
Comparison of the infectious fraction, ${i},$ produced by the dSPIR, Padé-1 SPIR, Padé-2 SPIR, and SPIR models for a number of values of ${{{R}}}_{0}$ (cf. Figure 5). Note that the total infected fraction at any moment would comprise the sum of *p* and ${i}$ fractions.

Figure 11 presents a comparison of profiles of $i(t)$ which result from numerical solution of the dSPIR, Padé SPIR, and SPIR models. The values

27 $${D}_{p}=\frac{1}{\alpha }=5.1 \; \mathrm{ days}, {D}_{i}-{D}_{p}=\frac{1}{\gamma }=3.3\;\mathrm{ days}$$

relevant to COVID-19^35,36^ are used in all simulations with ${R}_{0}\stackrel{\scriptscriptstyle\mathrm{def}}={\beta }_{i}\left({D}_{i}-{D}_{p}\right)+{\beta }_{p}{D}_{p}$.

Additional properties of the proposed SPIR models can be established in a similar manner^51^ and will be explored in more detail elsewhere.

Finally, in situations where there are data to warrant it, one can relax the basic premise of the preceding discussion, namely that the dynamics of a system with S, I, R compartments will likely be close to the dynamics of a system with a step function as cumulative distribution $\mathcal{F}$ of infectious period (Figs. 3 and 4). In such situations (e.g. models by Anderson et al.^52^) a corresponding SIR-like model structure can be developed that employs the ODE ${{\varvec{i}}}^{{{\prime}}}\left({\varvec{t}}\right)=\frac{\alpha }{D}\left({R}_{0}s\left(t\right)-1\right)i(t)$ in place of Eq. (12), where the parameter $\alpha$ ($1\le \alpha \le 2)$ is associated with the sigmoidicity of $\mathcal{F}.$ A full development of that case is presented in a separate publication^53^.

## Conclusion

We have made a case for revisiting the standard SIR model that describes the spread of infectious disease epidemics. While that model features valuable insights, it also has fundamental shortcomings related to quantifying the spread of an epidemic, as detailed in the main text. Therefore, use of that model to manage an epidemic could have adverse consequences. A remedy to this problem is proposed in the form of the Padé SIR model structure, which retains all qualitative features of the standard SIR structure as well as its simplicity, yet mitigates its systematic errors. It is also noted that the remedy proposed is not confined to the standard SIR model, but is applicable to the numerous compartment-based epidemiological models that constitute SIR variants, a re-examination of which would be warranted. The tools developed here can be easily and transparently incorporated in related software for practitioners or researchers^44,54,55^. Related formulas, derived in the main text, can be used both for epidemiological data processing to guide decision making as well as for theoretical analysis to advance the mathematical theory of epidemics.

## Supplementary Information

Supplementary Information.
