# Supplementary material for: Revisiting the standard for modeling the spread of infectious diseases
Source: Sci Rep. 2022 Apr 30;12:7077. doi: 10.1038/s41598-022-10185-0 (PMC9056532; doi:10.1038/s41598-022-10185-0)
Supplement: Supplementary file 1 — Supplementary Information. [file 41598_2022_10185_MOESM1_ESM.docx]

Revisiting the Standard for Modeling the Spread of Infectious Diseases

Michael Nikolaou*

Chemical and Biomolecular Engineering Department

University of Houston

Houston, TX 77204-4004

USA

*Corresponding author: Michael Nikolaou

Present address:

Chemical & Biomolecular Engineering Department

University of Houston

4226 MLK Blvd

Houston TX 77204-4004

USA.
Tel: +1 713 7434309

ORCID: 0000-0003-0241-6600

E-mail address: Nikolaou@uh.edu

**Supplementary material**

**APPENDIX A. Proof of eqn. (7)**

Following Figure 2, balance on $r_{k}, k=1, 2, 3,\ldots$ yields

$$r_{k}=\left( s_{k-1}-s_{k} \right)\mathcal{F}\left( 0 \right)+$$

$$+\left( s_{k-2}-s_{k-1} \right)\mathcal{F}\left( \delta t \right)+$$

$$+\left( s_{k-3}-s_{k-2} \right)\mathcal{F}\left( 2\delta t \right)+$$

$$\vdots$$

$$+\left( s_{0}-s_{1} \right)\mathcal{F}\left( \left( k-1 \right)\delta t \right)+$$

$$+\left( 1-r_{0}-s_{0} \right)\mathcal{F}\left( k\delta t \right)$$

$$+r_{0}$$

where $\mathcal{F}(t)$ is the cumulative distribution of discharge time from the I compartment the next compartment. Taking the limit for $\delta t\to0$ yields

$$r\left( t \right)=r\left( 0 \right)+\left( 1-r\left( 0 \right)-s\left( 0 \right) \right)\mathcal{F}\left( t \right)+\int_{0}^{t} \frac{d}{d\theta}\left[ s\left( t-\theta\right) \right]\mathcal{F}\left( \theta\right)d\theta=$$

$$=r\left( 0 \right)+\left( 1-r\left( 0 \right)-s\left( 0 \right) \right)\mathcal{F}\left( t \right)+\left. s\left( t-\theta\right)\mathcal{F}\left( \theta\right) \right|_{0}^{t}-\int_{0}^{t} s\left( t-\theta\right)\mathcal{F}^{'}\left( \theta\right)d\theta=$$

$$=r\left( 0 \right)+\left( 1-r\left( 0 \right)-s\left( 0 \right) \right)\mathcal{F}\left( t \right)+s\left( 0 \right)\mathcal{F}\left( t \right)-s\left( t \right)\underset{=0}{\underbrace{\mathcal{F}\left( 0 \right)}}-\int_{0}^{t} s\left( t-\theta\right)\underset{\mathcal{f}\left( \theta\right)}{\underbrace{\mathcal{F}^{'}\left( \theta\right)}}d\theta=$$

$$=r\left( 0 \right)+\left( 1-r\left( 0 \right) \right)\mathcal{F}\left( t \right)-\int_{0}^{t} s\left( t-\theta\right)\mathcal{f}\left( \theta\right)d\theta\Rightarrow$$

$$\boxed{r\left( t \right)=r\left( 0 \right)+\left( 1-r\left( 0 \right) \right)\mathcal{F}\left( t \right)-\int_{0}^{t} s\left( \theta\right)\mathcal{f}\left( t-\theta\right)d\theta}$$

$$=r\left( 0 \right)+\left( 1-r\left( 0 \right) \right)\mathcal{F}\left( t \right)-\int_{0}^{t} s\left( t-\theta\right)\mathcal{f}\left( \theta\right)d\theta\Rightarrow$$

$$\boxed{r\left( t \right)=r\left( 0 \right)+\int_{0}^{t} \underset{\Delta s\left( t-\theta\right)}{\underbrace{\left( 1-r\left( 0 \right)-s\left( t-\theta\right) \right)}}\mathcal{f}\left( \theta\right)d\theta}$$

Use of the Leibniz formula

$$\frac{d}{dx}\left( \int_{a\left( x \right)}^{b\left( x \right)} f\left( x,t \right)dt \right)= \mathcal{f}\left( x,b\left( x \right) \right)\frac{db}{dx} -\mathcal{f}\left( x,a\left( x \right) \right)\frac{da}{dx}+\int_{a\left( x \right)}^{b\left( x \right)} \frac{\partial}{\partial x}\mathcal{f}\left( x,t \right)dt$$

for $\mathcal{f}\left( \theta\right)=\lambda e^{-\lambda\theta}\Rightarrow$

$$r\left( t \right)=r\left( 0 \right)+\int_{0}^{t} \underset{\Delta s\left( t-\theta\right)}{\underbrace{\left( 1-r\left( 0 \right)-s\left( t-\theta\right) \right)}}\mathcal{f}\left( \theta\right)d\theta$$

$$=r\left( 0 \right)+\int_{0}^{t} \underset{\Delta s\left( \theta\right)}{\underbrace{\left( 1-r\left( 0 \right)-s\left( \theta\right) \right)}}\mathcal{f}\left( t-\theta\right)d\theta$$

$$=r\left( 0 \right)+\int_{0}^{t} \underset{\Delta s\left( \theta\right)}{\underbrace{\left( 1-r\left( 0 \right)-s\left( \theta\right) \right)}}\lambda e^{-\lambda\left( t-\theta\right)}d\theta\Rightarrow$$

$$r^{'}\left( t \right)=\underset{\Delta s\left( t \right)}{\underbrace{\left( 1-r\left( 0 \right)-s\left( t \right) \right)}}\lambda e^{-\lambda\left( t-t \right)}+\int_{0}^{t} \frac{\partial}{\partial t}\underset{\Delta s\left( \theta\right)}{\underbrace{\left( 1-r\left( 0 \right)-s\left( \theta\right) \right)}}\lambda e^{-\lambda\left( t-\theta\right)} d\theta$$

$$=\underset{\Delta s\left( t \right)}{\underbrace{\left( 1-r\left( 0 \right)-s\left( t \right) \right)}}\lambda+\int_{0}^{t} \frac{\partial}{\partial t}\underset{\Delta s\left( \theta\right)}{\underbrace{\left( 1-r\left( 0 \right)-s\left( \theta\right) \right)}}\lambda e^{-\lambda\left( t-\theta\right)} d\theta$$

$$=\underset{\Delta s\left( t \right)}{\underbrace{\left( 1-r\left( 0 \right)-s\left( t \right) \right)}}\lambda+\lambda\int_{0}^{t} \underset{\Delta s\left( \theta\right)}{\underbrace{\left( 1-r\left( 0 \right)-s\left( \theta\right) \right)}}\left( -\lambda\right)e^{-\lambda\left( t-\theta\right)} d\theta$$

$$=\underset{\Delta s\left( t \right)}{\underbrace{\left( 1-r\left( 0 \right)-s\left( t \right) \right)}}\lambda-\lambda\left( r\left( t \right)-r\left( 0 \right) \right)=\lambda i(t)$$

For step distribution $F$

$$\mathcal{f}\left( \theta\right)=\delta\left( \theta-D \right)\Rightarrow$$

$$r\left( t \right)=r\left( 0 \right)+\int_{0}^{t} \underset{\Delta s\left( t-\theta\right)}{\underbrace{\left( 1-r\left( 0 \right)-s\left( t-\theta\right) \right)}}\delta\left( \theta-D \right)d\theta=$$

$$=r\left( 0 \right)+\left( 1-r\left( 0 \right)-s\left( t-D \right) \right)H\left( t-D \right)$$

**APPENDIX B. Distribution functions in eqn. (7)**

To derive the formulas for $\mathcal{F(}\theta)$ in Figure 3, note that the inverse Laplace transform of $\tilde{\mathcal{F}}\left( q \right)=\frac{1}{q\left( \frac{1}{n\gamma}q+1 \right)^{n}}$ is the response of multiple first-order capacities in series to a unit-step input, which is well known to be $1-\frac{\Gamma\left( n, n\gamma\theta\right)}{\Gamma(n, 0)}$ where $\Gamma\left( a,z \right)=\int_{z}^{\infty} t^{a-1}e^{-t}dt.$

**APPENDIX C. Proof of eqn. (12)**

Define the variable

|  | $u=s-\bar{s}≝s-s\left( t<0 \right)$ | (28) |
| --- | --- | --- |

which trivially satisfies $u\left( t \right)=0$ for $t<0$, for simple application of the Translation Theorem of Laplace transforms. Then, take Laplace transforms, $\mathcal{L,}$ of eqn. (10) with $I\left( q \right)\mathcal{≝L}\left[ i\left( t \right) \right],$ $U\left( q \right)\mathcal{≝L}\left[ u\left( t \right) \right],$ to get

|  | $I(q)=\left( e^{-qD}-1 \right)U(q)$ | (29) |
| --- | --- | --- |

The main idea of creating SIR model approximations corresponding to ODE rather than DDE is to approximate the term $e^{-qD}$ in the above equation by a polynomial fraction, using Padé approximation of order 1 or higher. For example, using the second-order approximation

|  | $e^{-qD}\approx\frac{1-\frac{D}{2}q+\frac{D^{2}}{12}q^{2}}{1+\frac{D}{2}q+\frac{D^{2}}{12}q^{2}}$ | (30) |
| --- | --- | --- |

in eqn. (29) yields $I\left( q \right)=\frac{-Dq}{1+\frac{D}{2}q+\frac{D^{2}}{12}q^{2}}U\left( q \right)\Rightarrow\left( 1+\frac{D}{2}q+\frac{D^{2}}{12}q^{2} \right)I\left( q \right)=-DqU\left( q \right)\Rightarrow$

|  | $i\left( t \right)+\frac{D}{2}i^{'}\left( t \right)+\frac{D^{2}}{12}i^{''}(t)=-Du^{'}(t)=-Ds^{'}(t)=D\beta s(t)i(t)$ | (31) |
| --- | --- | --- |

which is eqn. (13).

Eqn. (12) can be proved similarly by showing that substituting $e^{-qD}$ by $\frac{1-\frac{D}{2}q}{1+\frac{D}{2}q}$ in eqn. (29) yields

|  | $i(t)+\frac{D}{2}i^{'}(t)=-Ds^{'}(t)=D\beta s(t)i(t)$ | (32) |
| --- | --- | --- |

Finally, to prove the equations in Figure 9, observe that if $s(0)$ changes from its previous value of 1 (assuming $r\left( 0 \right)=0$) by a step, $-\epsilon,$ then $U\left( q \right)=-\epsilon/q.$ Because $r\left( t\leq0 \right)=0$ and $s+i+r=1,$ it follows that $i$ jumps from $i\left( t<0 \right)=0$ to $i\left( 0 \right)=\epsilon.$ Therefore, the above eqn. (31) yields

|  | $\left( 1+\frac{D}{2}q+\frac{D^{2}}{12}q^{2} \right)I(t)=D\epsilon\Rightarrow\frac{i\left( \theta\right)}{\epsilon}\approx\mathcal{L}^{-1}\left[ \frac{D}{1+{qD}/2+{q^{2}D^{2}}/{12}} \right]=4\sqrt{3}ⅇ^{\frac{-3\theta}{D}}\sin\left( \frac{\sqrt{3}\theta}{D} \right)$ | (33) |
| --- | --- | --- |

Similarly

|  | $\left( 1+\frac{D}{2}q \right)I(t)=D\epsilon\Rightarrow\frac{i\left( \theta\right)}{\epsilon}\approx\mathcal{L}^{-1}\left[ \frac{D}{1+{qD}/2} \right]=2e^{\frac{-2\theta}{D}}$ | (34) |
| --- | --- | --- |

**APPENDIX D. Stability of dSIR and Padé SIR models**

**Stability of dSIR model.** Eqns. (9)-(11) imply that $\left( \bar{s},0, 1-\bar{s} \right)$ is an equilibrium point, where $\bar{s}$ can be arbitrary between $0$ and $1$. Then, local stability analysis of eqn. (9) by approximate linearization around the steady state $s_{-D}=s=\bar{s}$ yields

|  | ${\Delta s}^{'}(t)\approx-\left( \frac{\partial g}{\partial s} \right)_{\bar{s}}\Delta s(t)-\left( \frac{\partial g}{\partial s_{-D}} \right)_{\bar{s}}\Delta s(t-D)=\bar{s}\beta\Delta s(t)-\bar{s}\beta\Delta s(t-D)$ | (35) |
| --- | --- | --- |

where $\Delta s≝s-\bar{s},$ with $\Delta s\left( t<0 \right)=0, \Delta s\left( 0 \right)=\Delta s_{0}.$

Taking Laplace transforms with $\Delta S\left( q \right)\mathcal{≝L}\left[ \Delta s\left( t \right) \right]$ yields

|  | $q\Delta S(q)-\Delta s_{0}=\bar{s}\beta\Delta S(q)-\bar{s}\beta e^{-qD}\Delta S(q)\Rightarrow\Delta S(q)=\frac{1}{q-\bar{s}\beta+e^{-qD}\bar{s}\beta}\Delta s_{0}$ | (36) |
| --- | --- | --- |

The roots $p_{m}$ of the transcendental characteristic equation

|  | $q-\bar{s}\beta+e^{-qD}\bar{s}\beta=0$ | (37) |
| --- | --- | --- |

(poles of $\Delta S(q)$) can be obtained in terms of the Lambert function, $W,$ as follows: The last equation implies

$e^{qD-\bar{s}\beta D}\left( qD-\bar{s}\beta D \right)=-\bar{s}\beta Dⅇ^{-\bar{s}\beta D}\Rightarrow$ $p_{m}D-\bar{s}\beta D=W_{m}\left[ -\left( \bar{s}\beta D \right)ⅇ^{-\bar{s}\beta D} \right]\Rightarrow$

|  | $p_{m}=\bar{s}\beta\left( 1+\frac{W_{m}\left[ -(\bar{s}\beta D)ⅇ^{-\bar{s}\beta D} \right]}{\bar{s}\beta D} \right)$ | (38) |
| --- | --- | --- |

For stability, all $p_{m}$ must be in the left-half of the complex plane. We show first that for any positive value of $\bar{s},$ no complex root $p_{m}=\lambda+j\omega$ can cross the imaginary axis to move from stability to instability. Because, if $p_{m}=j\omega$ were a root of eqn. (37) for $\bar{s}>0,$ it would be

|  | $j\omega-\bar{s}\beta+e^{-j\omega d}\bar{s}\beta=0\Rightarrow\left\{ \begin{aligned} -\boxed{\bar{s}\beta}+\boxed{\bar{s}\beta}\cos\left( \omega D \right)=0\Rightarrow\omega D=2k\pi\\ \omega-\bar{s}\beta\sin\left( \omega D \right)=0\Rightarrow\omega=0 \end{aligned} \right\}$ | (39) |
| --- | --- | --- |

Therefore, only real roots should be considered in eqn. (38) for stability analysis. Furthermore, since

|  | $-e^{-1}\leq-ⅇ^{-\bar{s}\beta D}\bar{s}\beta D<0$ | (40) |
| --- | --- | --- |

the relevant values of $m$ are $0, -1$ in eqn. (38) for $p_{m}\mathbb{\in R}$, and $p_{m}<0$ implies

|  | $W_{m}\left( -xⅇ^{-x} \right)+x<0, m=0,-1$ $x≝\bar{s}\beta D$ | (41) |
| --- | --- | --- |

which is satisfied for $x<1$ (see figure below) leading immediately to eqn. (5).


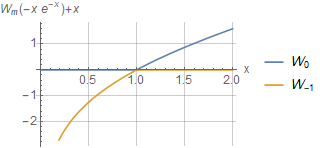


**Stability of Padé SIR models.** Approximate linearization of eqns. (12), (13), or, in general, any Padé approximation of order $n$, yields $\frac{d^{n}i}{dt^{n}}-a\left( R_{0}\bar{s}-1 \right)i\left( t \right)-b\approx0$ with $a>0$, e.g., $i^{'}\left( t \right)-\frac{2}{D}\left( R_{0}\bar{s}-1 \right)i\left( t \right)\approx0$ for Padé-1 SIR (eqn. (12)) or $i^{''}-\frac{12}{D^{2}}\left( R_{0}\bar{s}-1)i(t)-\frac{D}{2}i^{'}(t) \right)\approx0$ for Padé-2 SIR (eqn. (13)). The Routh-Hurwitz criterion immediately guarantees instability (spread of the epidemic) if $R_{0}\bar{s}-1>0,$ which is eqn. (5).

**APPENDIX E. Proof of eqn. (14)**

Dividing eqn. (9) by $s$ yields

|  | $\left( \ln s\left( t \right) \right)^{'}=-\beta\left( s\left( t-D \right)-s\left( t \right) \right)$ | (42) |
| --- | --- | --- |

Using eqn. (28) yields $s_{-D}-s=u_{-D}-u$. Then taking Laplace transforms, $\mathcal{L,}$ of both sides of eqn. (42) yields

$$\mathcal{L}\left[ \left( \ln s \right)^{'} \right]=\beta\left( 1-e^{-qD} \right)\mathcal{L}\left[ u \right]\Rightarrow$$

$$q\mathcal{L}\left[ \ln s \right]-\ln s_{0}=\beta\frac{\left( 1-e^{-qD} \right)}{q}q\mathcal{L}\left[ u \right]\Rightarrow$$

$$\lim_{q\to0} q\mathcal{L}\left[ \ln s \right]-\underset{\approx0}{\underbrace{\ln s_{0}}}=\beta\lim_{q\to0} \frac{\left( 1-e^{-qD} \right)}{q}\lim_{q\to0} q\mathcal{L}\left[ u \right]\Rightarrow$$

$$\ln s\left( \infty\right)-\ln s_{0}=\beta Du\left( \infty\right)$$

by the Final Value Theorem. Therefore

|  | $\frac{1}{\beta D}\ln s\left( \infty\right)-s\left( \infty\right)=\ln s_{0}-s_{0}\approx-1$ | (43) |
| --- | --- | --- |

for “small” values of $\epsilon,$ as shown in the following figure. (Recall that $s_{0}>\frac{1}{\beta D}\Leftrightarrow\epsilon<1-\frac{1}{\beta D}=\frac{1}{R_{0}}$ for the epidemic to spread.)


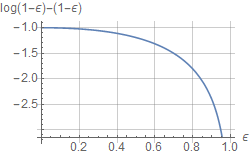


Continuing on the last equation we get

$$\left( -\beta Ds\left( \infty\right) \right)\exp\left( -\beta Ds\left( \infty\right) \right)=\left( -\beta D \right)\exp\left( -\beta D \right)\Rightarrow-\beta Ds\left( \infty\right)=W\left[ \left( -\beta D \right)\exp\left( -\beta D \right) \right]$$

which is eqn. (14).

The Padé-1 SIR model also reaches the same result: Dividing eqn. (1) by eqn. (12) and rearranging yields

$$\int_{s_{0}}^{s} \frac{\beta s-\gamma}{\beta s}ds=-2\beta\int_{i_{0}}^{i} di\Rightarrow s(t)-s\left( 0 \right)-\frac{\gamma}{\beta}\ln\left( \frac{s\left( t \right)}{s\left( 0 \right)} \right)=2\left( i\left( 0 \right)-i\left( t \right) \right)$$

Taking the limit as $t\to\infty$ with $1/\gamma=D,$ $s\left( 0 \right)=\bar{s},$ and $i\left( 0 \right)=\epsilon\approx0$ yields

$$s\left( \infty\right)=\frac{W[-ⅇ^{-R_{0}\bar{s}}R_{0}\bar{s}]}{R_{0}}$$

eqn. (14).

Proof that the standard SIR model also reaches the same result follows the same pattern and is omitted for brevity.

**APPENDIX** F**. Proof of eqns. (15)-(18)**

Using the Residue Theorem for Laplace transforms, eqn. (36) implies

|  | $\Delta s=\mathcal{L}^{-1}\left[ \Delta S \right]=\Delta s_{0}\sum_{m} e^{p_{m}t}\lim_{q\to p_{m}}\left[ \frac{q-p_{m}}{q-\bar{s}\beta+e^{-qD}\bar{s}\beta} \right]$ | (44) |
| --- | --- | --- |

where $p_{m}$ are the poles of $\Delta S(q)$, as shown in eqn. (38). With $\bar{s}\beta D>1$ (spreading epidemic) the argument $-(\bar{s}\beta D)ⅇ^{-\bar{s}\beta D}$ of $W_{m}$ in eqn. (38) satisfies the inequality in eqn. (40). Consequently, based on the properties of the Lambert function, the summation in eqn. (44) contains two terms with real poles, for $m=0,-1,$ with values

|  | $p_{-1}=\bar{s}\beta\left( 1+\frac{W_{-1}\left[ -(\bar{s}\beta D)ⅇ^{-\bar{s}\beta D} \right]}{\bar{s}\beta D} \right)=0$ | (45) |
| --- | --- | --- |

(because $W_{-1}\left( -xe^{-x} \right)=-x$ for $x\geq1$) and

|  | $p_{0}=\bar{s}\beta\left( 1+\frac{W_{0}\left[ -(\bar{s}\beta D)ⅇ^{-\bar{s}\beta D} \right]}{\bar{s}\beta D} \right)$ | (46) |
| --- | --- | --- |

The remaining poles are complex and with negative real parts (see APPENDIX D) as shown in the following figure of $p_{m}D, m=-1, 0, 1, 2, \ldots$:


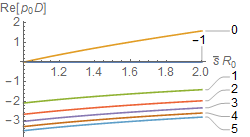


Therefore, the terms $e^{p_{m}t}$ rapidly decay for $m\geq1,$ and the summation in eqn. (44) quickly becomes approximately equal to

$$\Delta s(t)\approx\Delta s_{0}\left( \lim_{q\to0}\left[ \frac{q}{q-\bar{s}\beta+e^{-qD}\bar{s}\beta} \right]+e^{p_{0}t}\lim_{q\to p_{0}}\left[ \frac{q-p_{0}}{q-\bar{s}\beta+e^{-qD}\bar{s}\beta} \right] \right)\Rightarrow$$

|  | $\Delta s\left( t \right)\approx\Delta s_{0}\left( \frac{1}{1-\bar{s}\beta D}+e^{p_{0}t}\frac{1}{1-e^{-p_{0}D}\bar{s}\beta D} \right)$ | (47) |
| --- | --- | --- |

The time dependence of $i$ can be obtained in a similar fashion, as eqn. (10), combined with eqn. (36) implies

|  | $i\left( t \right)=\Delta s\left( t-D \right)-\Delta s\left( t \right)\Rightarrow I(q)=\left( e^{-qD}-1 \right)\Delta S(q)=\frac{\left( e^{-qD}-1 \right)\Delta s_{0}}{q-\bar{s}\beta+e^{-qD}\bar{s}\beta}$ | (48) |
| --- | --- | --- |

which eventually yields

|  | $i\left( t \right)\approx i\left( 0 \right)\left( \frac{D}{\bar{s}\beta D-1}+e^{p_{0}(t-D)}\frac{D}{e^{-p_{0}D}\bar{s}\beta D-1} \right)$ | (49) |
| --- | --- | --- |

The rates for Padé SIR and standard SIR are obtained by standard ODE linearization analysis and omitted for brevity.

**APPENDIX G. Proof of eqns. (19), (20)**

At the peak value $i^{*}$ of $i\left( t \right)$, eqn. of the first-order Padé SIR model implies

|  | $s^{*}=\frac{1}{\beta D}$ | (50) |
| --- | --- | --- |

Dividing eqn. (1) by eqn. (12) and integrating yields

|  | $\frac{ds}{di}=\frac{-\beta s}{2\left( \beta s-\frac{1}{D} \right)}\Rightarrow s\left( t \right)-s\left( 0 \right)-\frac{1}{\beta D}\ln\frac{s\left( t \right)}{s\left( 0 \right)}=-\frac{i\left( t \right)-i\left( 0 \right)}{2}$ | (51) |
| --- | --- | --- |

For $\left( 0 \right)\approx0,$ $s\left( 0 \right)\approx\bar{s}$, combining eqn. (50) with the above yields

|  | $i^{*}=2\left( \frac{1}{R_{0}}\ln\left( R_{0} \right)-\frac{1}{R_{0}}+s\left( 0 \right) \right)\Leftrightarrow\beta D≝R_{0}=\frac{2W_{-1}\left( \frac{i^{*}-2s(0)}{2s\left( 0 \right)ⅇ} \right)}{i^{*}-2s(0)}$ | (52) |
| --- | --- | --- |

where $W_{-1}$ is the Lambert function of order $-1$, as $-e^{-1}<\frac{-2+i^{*}}{2ⅇ}<0$ for $0\leq i^{*}\leq1,$ and $W_{0}\left( \frac{i^{*}-2s(0)}{2s\left( 0 \right)ⅇ} \right)$ does not yield feasible values above $1.$

The peak value $i^{*}$ of $i\left( t \right)$ for the standard SIR model is obtained in an entirely similar way and is omitted for brevity.

**APPENDIX H. SPIR model structure**

Corresponding flows from one SPIR compartment to another are shown in the following Figure S - 1 and in Figure S - 2 which is the counterpart of Figure 2.

| 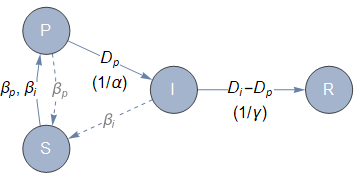 |
| --- |
| *Figure S - 1. The SPIR and dSPIR model structure, with members of the pre-symptomatic infectious compartment, P, moving, after time* $D_{p},$ *to the symptomatic infectious compartment, I, and from there, after time* $D_{i}-D_{p},$ *to the removed compartment, R. Both P and I infect the susceptible group, S, at rates,* $\beta_{p}$ *and* $\beta_{i},$ *respectively.* |

| \| $s_{-1}$ \| $s_{0}$ \| $s_{k-n_{2}}$ \| $\ldots$ \| $s_{k-n_{1}}$ \| $\ldots$ \| \| $s_{k-1}$ \| $s_{k}$ \| $s_{k+1}$ \| $\ldots$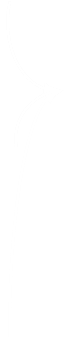 \| $s_{\infty}$ \| \| --- \| --- \| --- \| --- \| --- \| --- \| --- \| --- \| --- \| --- \| --- \| --- \| \|  \| … \| $p_{\infty}$ \| \|  \|  \|  \|  \| \|  \|  \| … \| … \|  \| \|  \| $p_{k}$ \| … \|  \| $i_{\infty}$ \| \|  \| \| $p_{k-1}$ \|  \|  \|  \|  \| \|  \| … \| \|  \|  \|  \|  \|  \| \|  \| $p_{k-n_{1}}$ \|  \| \|  \| $i_{k}$ \|  \|  \|  \| \|  \|  \|  \|  \| \|  \|  \|  \|  \|  \| \| $p_{0}$ \| $p_{k-n_{2}}$ \| $\ldots$ \| $i_{k-n_{1}}$ \| $\ldots$ \| \|  \| $\ldots$ \| $\ldots$ \| $\ldots$ \| $\ldots$ \| \| $r_{-1}$ \| $r_{0}$ \| $r_{k-n_{2}}$ \| $\ldots$ \| $r_{k-n_{1}}$ \| $\ldots$ \| \| $r_{k-1}$ \| $r_{k}$ \| $\ldots$ \| $\ldots$ \| $r_{\infty}$ \| \| $-1$ \| $0$ \| $1$ \| … \| $k-n_{1}$ \| \| … \| $k-1$ \| $k$ \| … \| … \| …$\infty$ \| |
| --- | --- | --- | --- | --- | --- | --- | --- | --- | --- | --- | --- | --- | --- | --- | --- | --- | --- | --- | --- | --- | --- | --- | --- | --- | --- | --- | --- | --- | --- | --- | --- | --- | --- | --- | --- | --- | --- | --- | --- | --- | --- | --- | --- | --- | --- | --- | --- | --- | --- | --- | --- | --- | --- | --- | --- | --- | --- | --- | --- | --- | --- | --- | --- | --- | --- | --- | --- | --- | --- | --- | --- | --- | --- | --- | --- | --- | --- | --- | --- | --- | --- | --- | --- | --- | --- | --- | --- | --- | --- | --- | --- | --- | --- | --- | --- | --- | --- | --- | --- |
| *Figure S - 2. Schematic of evolving susceptible (green), pre-symptomatic infectious (red), symptomatic infectious (orange), and removed (blue) fractions of a fixed-size population after an initial infection,* $p_{0}$*. Each new part of a fraction (thick-black bordered rectangles) moves to the next fraction (thick-black bordered rectangles to the right) in the same number of time steps. The population eventually reaches a steady state at* $s_{\infty}, r_{\infty}=1-s_{\infty}, i_{\infty}=p_{\infty}=0$*.* |
